# Toward the correction of effective electrostatic forces in explicit-solvent molecular dynamics simulations: restraints on solvent-generated electrostatic potential and solvent polarization

**DOI:** 10.1007/s00214-014-1600-8

**Published:** 2015-01-10

**Authors:** Maria M. Reif, Chris Oostenbrink

**Affiliations:** Institute for Molecular Modeling and Simulation, University of Natural Resources and Life Sciences, Vienna, Muthgasse 18, 1190 Vienna, Austria

**Keywords:** Computer simulation, Molecular dynamics, Electrostatic artifacts, Ion solvation, Solvent polarization

## Abstract

Despite considerable advances in computing power, atomistic simulations under nonperiodic boundary conditions, with Coulombic electrostatic interactions and in systems large enough to reduce finite-size associated errors in thermodynamic quantities to within the thermal energy, are still not affordable. As a result, periodic boundary conditions, systems of microscopic size and effective electrostatic interaction functions are frequently resorted to. Ensuing artifacts in thermodynamic quantities are nowadays routinely corrected a posteriori, but the underlying configurational sampling still descends from spurious forces. The present study addresses this problem through the introduction of on-the-fly corrections to the physical forces during an atomistic molecular dynamics simulation. Two different approaches are suggested, where the force corrections are derived from special potential energy terms. In the first approach, the solvent-generated electrostatic potential sampled at a given atom site is restrained to a target value involving corrections for electrostatic artifacts. In the second approach, the long-range regime of the solvent polarization around a given atom site is restrained to the Born polarization, i.e., the solvent polarization corresponding to the ideal situation of a macroscopic system under nonperiodic boundary conditions and governed by Coulombic electrostatic interactions. The restraints are applied to the explicit-water simulation of a hydrated sodium ion, and the effect of the restraints on the structural and energetic properties of the solvent is illustrated. Furthermore, by means of the calculation of the charging free energy of a hydrated sodium ion, it is shown how the electrostatic potential restraint translates into the on-the-fly consideration of the corresponding free-energy correction terms. It is discussed how the restraints can be generalized to situations involving several solute particles. Although the present study considers a very simple system only, it is an important step toward the on-the-fly elimination of finite-size and approximate-electrostatic artifacts during atomistic molecular dynamics simulations.

**Electronic supplementary material:**

The online version of this article (doi:10.1007/s00214-014-1600-8) contains supplementary material, which is available to authorized users.

## Introduction

Many processes of interest to (bio-)chemists take place in solution. They may be studied at an atomistic level using molecular simulation, where solvation effects are usually modeled explicitly via the presence of atomistic (fine-grained) solvent molecules or bead-like (coarse-grained) entities of solvent molecules, or implicitly via a potential of mean force approximation. The implicit-solvent approach, although computationally efficient, is fraught with limitations [1–4]. Thus, many studies dissuade from its use in simulations where an accurate representation of the solvent structure at short ranges from the solute is required. Despite numerous efforts to improve the description of short-range solvation by implicit-solvent models [5–10], the general consensus is to use a fine-grained explicit solvent representation whenever interfacial properties (e.g., solvent in the first-solvation shell of a solute) or the properties of individual solvent molecules (e.g., bridging solvent molecules in ligand–receptor interactions) are important. However, computation of the potential energy in a macroscopic system (on the order of $10^{23}$ particles) according to an interaction function incorporating all features necessary for a physically realistic description of a classical system (notably Coulombic electrostatic interactions, many-body interaction terms and electronic polarization) is not possible in an economic amount of time. Therefore, common simulation protocols at the time of writing imply systems of microscopic size (on the order of $10^4-10^6$ particles), pairwise effective electrostatic interactions functions and do not explicitly account for electronic polarization effects. Pairwise effective electrostatic interaction functions either rely on lattice summation [11–18] or cutoff truncation. Cutoff truncation may be done straightly without any additional modification of the Coulombic functional form, [19, 20] with additional ad hoc modifications of the functional form via switching, [21–29] shifting [21–24, 26, 28–41] or damping, [42–47] or with additional physics-based modifications in the form of a reaction-field correction [46, 48–53].

Due to their approximate nature, the effective interaction functions (e.g., lattice summation or cutoff schemes) introduce artifacts in the properties of various systems containing dipolar or charged particles, as recently reviewed extensively [54–60]. In the case of single-ion solvation, it is well understood how the incurred structural artifacts (i.e., long-range orientational solvent polarization deviating from the ideal Born polarization) affect thermodynamic properties such as solvation free energy and free-energy derivatives. In addition, it is also well understood how these properties can be corrected in an a posteriori analysis of the sampled configurations [61–66]. The present study refers to the correction scheme of Hünenberger and coworkers [63–66]. In the case of a cutoff-truncated electrostatic interaction function applied under periodic boundary conditions, this scheme accounts for (1) the neglect of solvent polarization beyond the cutoff sphere of the ion ($\Delta A_{A_1}$); (2) the spurious impact of cutoff truncation on the solvent polarization within the cutoff sphere of the ion ($\Delta A_{A_2}$); (3) the spurious impact of artificial periodicity on the solvent polarization within the cutoff sphere of the ion ($\Delta A_{B}$); and (4) a spurious offset in the electrostatic potential at the ion site due to improper summation of the electrostatic potential ($\Delta A_{C_1}$). Thus, it puts simulated solvent-generated electrostatic potentials on a par with those appropriate for a macroscopic nonperiodic system with Coulombic electrostatic interactions. For simulations with a lattice-sum electrostatic interaction function, slightly different corrections amounting to values of the same order of magnitude have been presented [63, 64, 67, 68].

The correction scheme mentioned above has proven very useful in the past [65, 66, 69, 70]. Concerning future developments, it is likely that increases in computational efficiency [71–73] and advances in multiscale simulation methodologies [74–80] will, in the long run, allow for the simulation of macroscopic nonperiodic systems with Coulombic electrostatic interactions, or electrostatic interactions truncated at sufficiently large distances, such that the entire effective interaction range of ionic solutes is encompassed in the simulated system. Since electrostatic interactions are decaying extremely slowly, this range is vast and extends to about 34 nm for a sodium ion in water [58]. At present, simulations of systems of this size are out of reach. Therefore, efforts to accurately model the interactions of charged particles with their surroundings in explicit-water molecular simulations have to be channeled into modifications of configurational sampling via on-the-fly application of corrective potentials. In this study, two possible alternative approaches are presented and illustrated for a single ion in water sampled with molecular dynamics (MD). In the first approach, the solvent-generated electrostatic potential sampled at the ion site is restrained to a target value that is corrected for artifacts intrinsic to the employed effective electrostatic interaction function. However, here only corrections acting within the cutoff sphere of the ion are included, because the restraint forces only act on water molecules within the cutoff sphere. Nevertheless, the authors of this study consider the restraint Ansatz a viable first attempt to address electrostatic artifacts on-the-fly. In the second approach, the long-range radial orientational solvent polarization around the ion is restrained to the Born polarization. The associated special potential energy and force terms can be easily integrated into a MD algorithm. Because of the radial symmetry of the system, implementation for simulations involving a single ionic solute is relatively straightforward as the required target values (average electrostatic potential or polarization) are constant throughout the simulation.

**Fig. 1:**
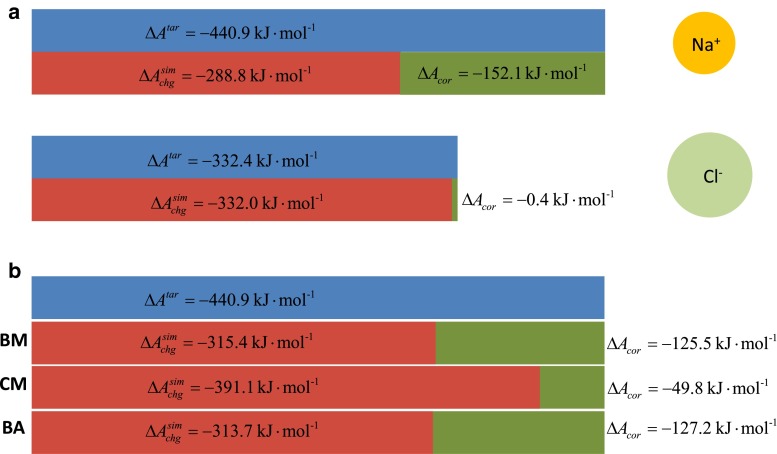
Effect of applying finite-size and approximate-electrostatics corrections [63, 64] to the charging free energies of cationic and anionic molecules, illustrated for the case of sodium and chloride ions with effective radii of [65] $R_I=0.168$ and 0.246 nm, respectively, and with Lennard-Jones parameters according to the GROMOS 54A8 force field [65, 66] in combination with the SPC water model [159]. **a** The charging free energies of the infinitely dilute ions in a macroscopic nonperiodic system with Coulombic electrostatic interactions are given by $\Delta A_{\rm {tar}}$. For the spurious simulated situation of the BM scheme under periodic boundary conditions in a cubic computational box with $R_C=1.4$ nm, $\epsilon _{BW}=66.6$ and $L=4.04$ nm, the charging free energies evaluate to $\Delta A_{\rm{chg}}^{\rm {sim}}$. The correction terms, evaluated according to Ref. [64] are $\Delta A_{A_1}=-48.9$, $\Delta A_{A_2}=-24.7$, $\Delta A_{B}=-1.9$, $\Delta A_{C_1}=-75.7$ and $\Delta A_{D}=-0.9\, \hbox {kJ}\;\hbox {mol}^{-1}$ for the sodium ion and $\Delta A_{A_1}=-48.9$, $\Delta A_{A_2}=-24.6$, $\Delta A_{B}=-1.7$, $\Delta A_{C_1}=75.4$ and $\Delta A_{D}=-0.6\, \hbox {kJ}\;\hbox {mol}^{-1}$ for the chloride ion, where the fitted functions described in Ref. [64] were used for $\Delta A_{A_2}$ and $\Delta A_B$. **b** The magnitude of the overall correction term is reduced by $\Delta A_{A_2}$ and $\Delta A_B$ if an electrostatic potential restraint involving these two corrections is used. For the example of sodium ion hydration, these two quantities evaluate to [63, 64] $\Delta A_{A_2}=-24.7$ or $15.5\, \hbox {kJ}\;\hbox {mol}^{-1}$ and $\Delta A_{B}=-1.9$ or $-0.6\, \hbox {kJ}\;\hbox {mol}^{-1}$ for the schemes with reaction-field correction (BM, BA) or the CM scheme, respectively. The correction term $\Delta A_{{cor}}$ for the BM scheme thus amounts to $-125.5\, \hbox {kJ}\;\hbox {mol}^{-1}$. Its contributions ($\Delta A_{A_1}$, $\Delta A_{C_1}$, $\Delta A_D$) are reported in (**a**). For the CM scheme, $\Delta A_{ cor }$ has contributions from $\Delta A_{A_1}$ and $\Delta A_D$ ($-48.9$ and $-0.9\,\hbox {kJ}\; \hbox {mol}^{-1}$, respectively) and for the BA scheme, it has contributions from $\Delta A_{A_1}$, $\Delta A_{C_1}$ and $\Delta A_D$ ($-48.9$, $-77.4$ and $-0.9\, \hbox {kJ}\;\hbox {mol}^{-1},$ respectively)

In principle, generalization to the case of multiple solutes is possible. Future work will explore the application of the two restraints to the calculation of an ion–ion potential of mean force. It has been suggested before [65, 66] that ion–ion potentials of mean force in water, i.e., the free energy describing the reversible association–dissociation equilibrium of two hydrated ions, calculated with an approximate-electrostatic interaction function, are afflicted by errors due to the underhydration of cations when their ion–water Lennard-Jones parameters were calibrated against methodology-independent hydration free energies. This is because for cations, the correction terms converting a methodology-dependent hydration free energy to the corresponding methodology-independent value are negative and of large magnitude. Consider, for instance, the hydration of a sodium ion in Fig. 1a. The ion was parameterized such that the methodologically independent solvation contribution due to the free energy of charging the ion matches the target value of $-440.9\,\hbox {kJ}\; \hbox {mol}^{-1}$. This value is exempt of contributions for air–water interface crossing, cavity formation and standard-state conversion (i.e., this value refers to identical ion concentrations in air and in water) [58]. If all three of the latter contributions were added, one could compare the resulting number to an experimental real hydration free energy and if only the last two were added, one could compare the resulting number to an experimental intrinsic hydration free energy (based on a standard intrinsic proton hydration free energy of $-1{,}100\,\hbox {kJ}\; \hbox {mol}^{-1}$) [58]. In theoretical work, e.g., using a cubic box with edge length 4.04 nm, molecule-based cutoff truncation at a distance of 1.4 nm for electrostatic interactions, as well as a reaction-field correction for omitted electrostatic interactions, the calculated value ($-440.9\,\hbox {kJ}\;\hbox {mol}^{-1}$) is obtained from two components: a “raw” charging free energy of $-288.8\, \hbox {kJ}\; \hbox {mol}^{-1}$ that is deduced from a computer simulation and another $-152.1\,\hbox {kJ}\; \hbox {mol}^{-1}$ from the indicated corrections that are added manually in post-simulation work. However, this means that the underlying sampling during the simulation (and hence the forces) corresponds to an ion with a charging free energy of $-288.8\,\hbox {kJ}\; \hbox {mol}^{-1}$. Hence, a large part of the actual hydrophilicity of the cation is not accounted for in simulations that are performed in the “usual” way, i.e., in microscopic or periodic systems and with electrostatic interactions that are not strictly Coulombic. As a consequence, the interaction of cations with species other than water might be too favorable. On the contrary, for anions, the magnitude of the correction terms is not that large, because a considerable contribution due to the spurious summation of the electrostatic potential ($\Delta A_{C_1}$) is positive. This is because it is proportional to the ionic charge rather than to its square. Therefore, this contribution decreases the magnitude of the overall (negative) correction term (Fig. 1a). Note that these considerations only hold for the specific case of solvent molecules with a positive molecular quadrupole moment trace (e.g., the SPC water model) and for simulations carried out with an effective electrostatic interaction function involving this particular summation artifact [68].

This paper is organized as follows: Sect. 2 describes the theoretical framework of the performed simulations and introduces the electrostatic potential and polarization restraints. Simulation details are reported in Sect. 3. Section 4 shows how structural features of the solvent and the thermodynamics of ion–solvent and solvent–solvent interactions are influenced by either of these restraints. Finally, Sect. 5 provides concluding remarks and an outlook.

## Theory

The following section offers a rationalization for using a cutoff scheme in the current work. The two subsequent sections introduce the electrostatic potential and polarization restraints. These sections consider a system consisting of a single ion in water. For simplicity, it is assumed that the ion is spherical. Section 2.4 will discuss extension of the methodology to systems with non-spherical symmetry, e.g., a hydrated oligoatomic ion, multiple hydrated ions or ions in heterogeneous environments. Throughout, angular brackets ($\langle \cdot \cdot \cdot \rangle$) refer to plain time (ensemble) averaging, whereas, unless stated otherwise, overlines ($\overline{\cdot \cdot \cdot }$) indicate an alternative calculation of average properties.

### Lattice-sum versus cutoff-truncation electrostatic interaction functions

The current work focuses on approximate-electrostatic interaction schemes employing a cutoff. Note, however, that lattice-sum methods are not exempt of artifacts either, which can also be corrected a posteriori [18, 35, 63, 67]. In a simulation using a lattice-sum method, performed as in the example above, in a cubic box with edge length 4.04 nm, the charging free energy of $-440.9\,\hbox {kJ}\;\hbox {mol}^{-1}$ of the sodium ion is built up of a raw charging free energy of $-314.5\,\hbox {kJ}\;\hbox {mol}^{-1}$ and correction terms amounting to $-126.4\,\hbox {kJ}\;\hbox {mol}^{-1}$. Hence, although the nature of the artifacts introduced by lattice-sum methods may be different, they are of comparable size [63]. Due to the widespread use of lattice-sum methods, a brief digression into the associated artifacts is done here in order to (1) explain those artifacts; (2) discuss their impact on the sampled configurations in MD simulations of solution-phase systems; (3) compare the benefits and shortcomings of cutoff-truncated and lattice-sum electrostatic interaction functions; and (4) explain why the former electrostatic interaction scheme was chosen to illustrate the main idea of this paper. The discussion will be biased toward the hydration of single ions because this is the main topic of the present work.

1. The water polarization around a single hydrated ion, as obtained from a MD simulation with a lattice-sum electrostatic interaction function, shows one major artifact in comparison with an ion at infinite dilution: The polarization is too low because water molecules in the central computational box also interact with all the periodic copies of the ion [58, 63]. Of course, this phenomenon may be considered physical if one is actually interested in such a periodic system or in the solution of the given ion at the concentration $N_A^{-1}L^{-3}$, where $N_A$ is Avogadro’s constant and $L$ the edge length of the computational box (assumed to be a cube). The main focus of the present work is on ions at infinite dilution, and therefore, the periodicity of interactions in simulations with a lattice-sum electrostatic interaction function is a most undesired feature in the present context. One should, however, not forget that for $L\rightarrow \infty$, the periodicity artifacts incurred in simulations with a lattice-sum method vanish and the interactions become Coulombic. Since simulations in such huge systems (boxes of $\approx$ 80 nm edge length; see below) are currently out of reach, the authors still think that present-day lattice-sum simulations of ions at infinite dilution give spurious results.
  The latter artifact, i.e., an underestimated water polarization around ions, is of a structural nature but also propagates into thermodynamic properties, e.g., hydration free energies [58, 63]. It is a widely known and well-understood problem of simulations with a lattice-sum electrostatic interaction function [18, 35, 58, 63, 65, 67, 69, 70, 81, 82]. There is a second issue that is not reflected in structural properties but only in thermodynamic properties. It relates to the boundary conditions implied by a lattice-sum electrostatic interaction function. While in a lattice-sum simulation of a single hydrated ion the average of the electrostatic potential over the volume of the computational box evaluates to zero, in reality the zero of electrostatic potential should be located infinitely far away from the ion [68]. The resulting contribution to the hydration free energy is sizeable and does not vanish when $L\rightarrow \infty$. It is still controversial whether this free energy offset should be conceived physical or artificial [58, 68, 83–90]. Drawing the problem back to the question whether the hydrated ion should experience the internal or external Galvani potential of the solvent phase, [58, 68] and noting that ions of atomic size have an excluded volume, suggests that this free energy offset encountered in simulations with a lattice-sum electrostatic interaction function is artificial because it leads to the inclusion of “interior” solvent contributions (related to the quadrupole moment trace of the solvent model [58, 68, 91]) in the electrostatic potential at the ion site.
2. The discussion of the impact of a lattice-sum electrostatic interaction function on configurational sampling has to distinguish two cases. First, in the present situation of a single monoatomic ion in water, only the configurational sampling of the solvent is affected in that the water polarization is wrong [see point (1)]. Second, in the case of non-rigid polyatomic solutes, the configurational sampling of the solute itself will be affected if pronounced interactions between periodic solute copies occur [54, 92, 93]. Again, this is physical if the simulation of a crystalline system is intended, [94] but is spurious if one is actually interested in the solute at infinite dilution. The magnitude of the artifact decreases as $L \rightarrow \infty$.
3. In the view of the artifacts introduced by lattice-sum methods, three possible remedies may be thought of. First, one can simply increase the edge length of the computational box. Second, lattice-sum methods may be modified such that artificial periodicity is eliminated. This can e.g., be done by orientational averaging of the lattice-sum electrostatic potential [30, 36, 38–40] or combination of the lattice-sum interaction function with other nonperiodic functions [53, 95–99]. Third, one can resort to another electrostatic interaction function, e.g., one involving cutoff truncation. A tremendous amount of studies are dedicated to the comparison of different electrostatic interaction functions for the simulation of condensed-phase systems [29, 32, 34, 35, 51, 52, 54, 63, 94, 100–133] and the results (considering studies up to the year 2009) are summarized in Ref. [54] Clearly, lattice-sum methods are ideally suited for the simulation of periodic systems such as crystals. In addition, as the electrostatic interactions become Coulombic in the limit of infinite box edges, lattice-sum methods seem to be good for the simulation of systems in large computational boxes, possibly with a high ion strength to achieve screening of electrostatic interactions which further reduces the interactions between periodic solute copies. However, whenever the interaction between periodic solute copies is considered unphysical, interaction functions involving cutoff truncation offer the advantage of a user-defined tuning of the lengthscale of allowed interactions. Obviously, simply eliminating the long-range nature of electrostatic interactions introduces additional problems. In the case of ion hydration, these problems have so far only been addressed in the post-processing of simulation results. The present work, however, proposes a way to tackle these problems on-the-fly, i.e., on the level of the forces. As an aside, note that a second important advantage of cutoff schemes in comparison with lattice-sum schemes is that they are computationally cheaper.
4. The treatment of single solvated ions with either lattice-sum or cutoff schemes introduces errors in the solvent structure and in the thermodynamic characteristics of ion hydration. These errors have been discussed and compared very thoroughly by Hünenberger et al. [63–65, 134]. Given currently affordable computational expenses, the increase in the relevant parameters of the simulated system (box-edge length in the case of lattice-sum and cutoff methods) and of the electrostatic interaction function (cutoff length in the case of cutoff methods) required to render these errors negligible is not possible. For example, in lattice-sum simulations, the error in the hydration free energy of a single ion due to underpolarization of the solvent is [63, 67]
  1 $$\begin{aligned} \Delta A&= - (8 \pi \epsilon _o)^{-1} q_I^2 (1-\epsilon _S^{-1}) L^{-1} \left[ \xi + \frac{4 \pi }{3} \left( \frac{R_I}{L}\right) ^2 - \frac{ 16 \pi ^2}{45} \left( \frac{R_I}{L}\right) ^5 \right] , \end{aligned}$$
  where $\epsilon _o$ is the permittivity of vacuum, $q_I$ and $R_I$ are the charge and radius of the ion, respectively, $\epsilon _S$ the solvent permittivity and [35, 67, 135–137] $\xi \approx -2.837297$ a constant (valid for a cubic computational box). For $R_I \ll L$ and $\epsilon _S\rightarrow \infty,$ $\Delta A$ in Eq. 1 approaches $- (8 \pi \epsilon _o)^{-1} q_I^2 \xi L^{-1}$. In view of this limit, note that the error in the hydration free energy given by Eq. 1 is not to be confused with the interaction of the solute with its periodic copies (“self-term” [18, 53, 135, 137]). If the “self-term,” i.e., the electrostatic potential created by the periodic ion copies [$(4 \pi \epsilon _o)^{-1} q_I \xi L^{-1}$] was spuriously included in the electrostatic potential at the ion site (e.g., if the equivalent vacuum contribution is not removed), an additional error of $(8 \pi \epsilon _o)^{-1} q_I^2 \xi L^{-1}$ would occur in the hydration free energy of the ion. For $R_I \ll L$ and $\epsilon _S\rightarrow \infty$, this term counteracts the magnitude of Eq. 1 but does not correct the sampling of the solvent configurations. The value of $\Delta A$ is actually sizeable for usual box sizes up to 10 nm. One can see that e.g., for $q_I=1~e$ and $\epsilon _S=78.4$ (as appropriate for water), $\Delta A$ (Eq. 1) is smaller than $2.5\,\hbox {kJ}\;\hbox {mol}^{-1}$ (i.e., the thermal energy at 298.15 K) for $L>78$ nm. As a second example, in simulations using cutoff truncation of the electrostatic interactions, the error in the hydration free energy of a single ion due to the omitted solvent beyond the cutoff sphere is [63, 138, 139]
  2 $$\begin{aligned} \Delta A = (8 \pi \epsilon _o)^{-1} q_I^2 (1-\epsilon _S^{-1}) R_C^{-1}, \end{aligned}$$
  where $R_C$ is the cutoff distance. One can see that e.g., for $q_I=1~e$ and $\epsilon _S=78.4$, $\Delta A$ (Eq. 2) is smaller than $2.5\,\hbox {kJ}\;\hbox {mol}^{-1}$ for $R_C>27$ nm. Therefore, at present, both lattice-sum and cutoff methods seem “equally bad” when it comes to the simulation of single hydrated ions. As the latter method is computationally more efficient, it is the main concern of the present work.

### Electrostatic potential restraint

The electrostatic potential restraint allows simulation of an ion $I$ in explicit water under the condition that the average solvent-generated electrostatic potential sampled at the ion site be equal to a given value $\phi _{\rm {tar}}$. The instantaneous solvent-generated electrostatic potential at the ion site $\mathbf{r}_I$ at time $t$ is $\phi (t, \mathbf{r}_I; \mathbf{x})$, where $\mathbf{x}$ denotes the $3N$-dimensional coordinate vector of the system containing the ion and $N_s$ solvent molecules, each consisting of $N_{s,a}$ atoms. It is given as a sum over the pairwise interactions of the ion with charge $q_I$ with the solvent atoms $i$,

3 $$\begin{aligned} \phi (t, \mathbf{r}_I; {\mathbf{x}}{(t)} ) = (4 \pi \epsilon _o)^{-1} \sum _{i=1}^{N_sN_{s,a}} q_i \psi _{Ii}(\mathbf{x}(t)), \end{aligned}$$

where $\epsilon _o$ is the vacuum permittivity and $\psi _{Ii}(\mathbf{x}(t))$ is the pairwise electrostatic interaction function evaluated for sites $I$ and $i$ at time $t$. For example, for strictly Coulombic electrostatic interactions,

4 $$\begin{aligned} \psi _{Ii}(\mathbf{x}(t)) = \frac{1}{ \left| \mathbf{r}_I(t) - \mathbf{r}_i(t)\right| }, \end{aligned}$$

while for the approximate-electrostatic schemes different forms are used, namely

5 $$\begin{aligned} \psi _{Ii}(\mathbf{x}(t)) = \frac{1}{ \left| \mathbf{r}_I(t) - \mathbf{r}_i(t) \right| } - \frac{1}{R_C} \end{aligned}$$

for a straight truncation of electrostatic interactions at a cutoff distance $R_C$ and

6 $$\begin{aligned} \psi _{Ii}(\mathbf{x}(t)) = \frac{1}{ \left| \mathbf{r}_I(t) - \mathbf{r}_i(t) \right| } + \frac{\epsilon _{RF} -1}{2 \epsilon _{RF}+1} \frac{( \mathbf{r}_I(t) - \mathbf{r}_i(t) )^2}{R_C^3} - \frac{3 \epsilon _{RF}}{2 \epsilon _{RF}+1} \frac{1}{R_C} \end{aligned}$$

for a truncation of electrostatic interactions at a cutoff distance $R_C$ combined with a reaction-field correction deriving from a dielectric continuum of static relative dielectric permittivity $\epsilon _{RF}$ outside the cutoff sphere of each particle [48]. The cutoff truncation can be performed in an atom- or molecule-based fashion [140]. When applied under periodic boundary conditions, Eqs. 5 and 6 are altered to rely on minimum-image distances. To simplify the notation, $\phi (t, \mathbf{r}_I; \mathbf{x}(t))$ will be written as $\phi (t; \mathbf{r}_I)$ and can be evaluated as

7 $$\begin{aligned} \phi (t; \mathbf{r}_I) = q_I^{-1} E_I^{\rm {elec}}(t), \end{aligned}$$

where $E_I^{\rm {elec}}(t)$ is the electrostatic interaction energy between the ion and the solvent at time $t$. The time average of $\phi (t; \mathbf{r}_I)$, evaluated at time $t^{\prime}$, is the average over the sampled trajectory of length $t^{\prime}$,

8 $$\begin{aligned} \langle \phi (t^{\prime}; \mathbf{r}_I) \rangle = {t^{\prime}}^{-1} \int _{0}^{t^{\prime}} \hbox {d}t \, \phi (t; \mathbf{r}_I), \end{aligned}$$

and is required to be equal to the target value $\phi _{\rm {tar}}$. This can be enforced by application of a corresponding restraining potential, allowing e.g., harmonic deviations according to a force constant $k$ from the target value,

9 $$\begin{aligned} V^{\rm {restr}} ( \langle \phi (t; \mathbf{r}_I) \rangle; \phi _{\rm tar}, k ) = \frac{1}{2} k \left[ \langle \phi (t; \mathbf{r}_I) \rangle - \phi _ {\rm {tar}} \right] ^2. \end{aligned}$$

The choice of the target value $\phi _{\rm {tar}}$ is discussed in Sect. 2.4. Since the plain time average of the electrostatic potential, as given by Eq. 8 is not suitable to derive restraint forces by differentiating Eq. 9, $\langle \phi (t^{\prime}; \mathbf{r}_I) \rangle$ is estimated in terms of a decay time [141] $\tau$, such that the time average of the electrostatic potential sampled at the ion site at time $t^{\prime}$ becomes

10 $$\begin{aligned} \overline{\phi (t^{\prime}; \tau , \mathbf{r}_I)}&= \left\{ \tau \left( 1-\exp \left[ -\tau ^{-1} t^{\prime}\right] \right) \right\} ^{-1} \int _{0}^{t^{\prime}} \hbox {d}t \exp \left[ -\tau ^{-1} (t^{\prime}-t) \right] \phi (t; \mathbf{r}_I) \nonumber \\&= \left\{ \tau \left( 1-\exp \left[ -\tau ^{-1} t^{\prime}\right] \right) \right\} ^{-1} \int _{0}^{t^{\prime}} \hbox {d}t \exp \left[ -\tau ^{-1} (t^{\prime}-t) \right] q_I^{-1} {E}_I^{\rm {elec}}(t). \end{aligned}$$

Using the time relaxation formalism of Eq. 10, the restraining potential of Eq. 9 is formally rewritten as

11 $$\begin{aligned} V^{\rm {restr}} ( \overline{\phi (t; \tau , \mathbf{r}_I)} ; \phi _{\rm {tar}}, k ) = \frac{1}{2} k \left[ \overline{\phi (t; \tau , \mathbf{r}_I)} - \phi _{\rm {tar}} \right] ^2, \end{aligned}$$

and if the decay time is much smaller than the simulation length, i.e., $\tau \ll t$, the time average $\overline{\phi (t; \tau , \mathbf{r}_I)}$ given by Eq. 10 can be written in terms of discrete integration time steps $\Delta t$ as [141]

12 $$\begin{aligned} \overline{\phi (t; \tau , 
\mathbf{r}_I)} = \left( 1- \exp \left[ -\tau ^{-1} \Delta t\right] \right) \phi (t; \mathbf{r}_I) +\,\exp \left[ - \tau ^{-1} \Delta t\right] \overline{\phi (t - \Delta t; \tau , \mathbf{r}_I)}. \end{aligned}$$

Note that introduction of memory kernels in the equations of motion implies time-irreversible dynamics. This is not considered a problem here as the properties of interest (structural and thermodynamic properties of an ion-in-water system) are expected to be independent of the time-reversibility of the equations of motion. Besides, numerical noise [142–146] and commonly used implementations of constraints (e.g., for bond lengths) [147] in ordinary MD simulations already prevent the dynamics from being rigorously time reversible even if time-reversible integrators are used. Note, in addition, that the evaluation of Eq. 12 only involves three arithmetic operations per time step, i.e., evaluation of the time integral captured by $\overline{\phi (t; \tau , \mathbf{r}_I)}$ does not come with increases in computational cost as $t$ increases.

One can calculate the restraint forces $\mathbf{F}^{\rm {restr}}_I(t; \mathbf{x})$ acting on the ion and $\mathbf{F}^{\rm {restr}}_i(t; \mathbf{x})$ acting on solvent atom $i$,

13 $$\begin{aligned} \mathbf{F}^{\rm{restr}}_I(t; \mathbf{x}) = - \sum _{i=1}^{N_sN_{s,a}} \mathbf{F}^{\rm {restr}}_i(t; \mathbf{x}) \end{aligned}$$

by differentiating Eq. 11. The force $\mathbf{F}^{\rm {restr}}_I(t; \mathbf{x})$ is thus

14 $$\begin{aligned} \mathbf{F}^{\rm {restr}}_I(t; \mathbf{x})&= - \frac{\partial V^{\rm {restr}} ( \overline{\phi (t; \tau , \mathbf{r}_I)}; \phi _{\rm {tar}}, k) }{ \partial \mathbf{r}_I} \nonumber \\&= - k \left[ \overline{\phi (t; \tau , \mathbf{r}_I)} - \phi _{\rm {tar}} \right] \frac{ \partial \overline{\phi (t; \tau , \mathbf{r}_I)} }{ \partial \mathbf{r}_I}. \end{aligned}$$

Using Eq. 12 together with

15 $$\begin{aligned} \frac{\partial \phi (t; \tau , \mathbf{r}_I)}{\partial \mathbf{r}_I}=-q_I^{-1} \mathbf{F}_I^{\rm {elec}}(t), \end{aligned}$$

where $\mathbf{F}_I^{\rm {elec}}(t)$ is the electrostatic force exerted at time $t$ by the solvent on the ion, one finds

16 $$\begin{aligned} \frac{ \partial \overline{\phi (t; \tau , \mathbf{r}_I)} }{ \partial \mathbf{r}_I} = - \left( 1- \exp \left[ -\tau ^{-1} \Delta t\right] \right) q_I^{-1} \mathbf{F}_I^{\rm {elec}}(t) + \exp \left[ - \tau ^{-1} \Delta t \right] \frac{\partial \overline{\phi (t-\Delta t; \tau , \mathbf{r}_I)} }{\partial \mathbf{r}_I}, \end{aligned}$$

where the second term on the right-hand side vanishes because the average of the electrostatic potential at the previous time step does not depend on the coordinates at time $t$. Combining Eqs. 14 and 16 one gets

17 $$\begin{aligned} \mathbf{F}^{\rm {restr}}_I(t; \mathbf{r}_I) = k \left[ \overline{\phi (t; \tau , \mathbf{r}_I)} - \phi _{\rm {tar}} \right] \left( 1- \exp \left[ -\tau ^{-1} \Delta t\right] \right) q_I^{-1} \mathbf{F}_I^{\rm {elec}}(t). \end{aligned}$$

The calculation of the restraint force can thus be performed without any additional computational cost since the electrostatic interaction energies between the ion and the solvent, as well as the corresponding forces are available.

Note that at the start of the simulation, the average electrostatic potential of Eq. 10 is not defined, and is therefore set equal to the instantaneous (initial) electrostatic potential $\overline{\phi (t=0; \tau , \mathbf{r}_I)}= \phi (t=0; \mathbf{r}_I)$.

Note, in addition, that the autocorrelation function of the electrostatic potential decays sufficiently rapidly so that the time relaxation formalism of Eq. 10 is applicable here [141]. For instance, the autocorrelation function of the electrostatic potential sampled at e.g., the $\hbox {Na}^+$ ion in SPC water in the NPT ensemble at a pressure of $P^{\circ }=1$ bar and a temperature of 300 K decays within 3–4 ps.

In summary, application of the electrostatic potential restraint corrects the electrostatic potential sampled at the ion site by a certain amount. This propagates into the charging free energy of the ion, which is corrected by the corresponding free-energy contribution. The power of the electrostatic potential restraint thus resides in the translation of previously proposed [63] charging free energy corrections to on-the-fly corrections affecting the forces in a MD simulation. An example concerning the calculation of charging free energies is discussed in Sect. 2.4, and the results are presented in Sect.  4.1.

### Polarization restraint

For distances sufficiently far away from the ion, the radial polarization loses its solvent shell structure. In the ideal case of Coulombic electrostatic interactions in a nonperiodic, macroscopic system, it is equal to the Born polarization,

18 $$\begin{aligned} P_{\rm {Born}}(r)=\frac{q_I}{4\pi r^2}\left( 1-\frac{1}{\epsilon _S^{\prime }}\right) , \end{aligned}$$

where $\epsilon _S^{\prime}$ is the relative dielectric permittivity of the solvent model. However, this is not the case in practice when an effective electrostatic interaction scheme is employed. In the case of lattice summation, the radial polarization around the ion is underestimated. This underpolarization is, however, best corrected with the electrostatic potential restraint (Sect. 2.2), because restraining the polarization to the Born polarization is not a viable solution in the vicinity of the ion. In the case of cutoff truncation of electrostatic interactions, the electrostatic potential restraint (Sect. 2.2) acts only within the cutoff sphere, i.e., only corrects the polarization in a given range around the ion. The polarization right at the cutoff distance is still spuriously affected even if the electrostatic potential restraint is applied. This artifact can be eliminated by a restraint to the Born polarization.

The polarization restraint allows simulation of an ion $I$ in explicit water under the condition that the average polarization in a small distance range $[R^{\prime}- 0.5\Delta R_P; R^{\prime}+0.5 \Delta R_P]$ around the ion be equal to a target polarization $P_{\rm {tar}}(R^{\prime})$, where $R^{\prime}$ is the center of a small bin of width $\Delta R_P$. Here, $R^{\prime}$ is chosen to be within a certain distance interval from $R_1^{\rm {restr}}$ to $R_2^{\rm {restr}}>R_{1}^{\rm {restr}}$ from the ion, i.e., $R^{\prime}\in [R_1^{\rm {restr}}; R_2^{\rm {restr}}]$, the polarization restraints only being active within this interval.

The instantaneous polarization at a distance $R^{\prime}$ from the ion at time $t$ is $P(t; R^{\prime}, \mathbf{x})$, where $\mathbf{x}$ denotes the $3N$-dimensional coordinate vector of the system containing the ion and $N_s$ solvent molecules. It is given by

19 $$\begin{aligned} P(t; R^{\prime}, \mathbf{x})=\mu _S^{\prime}\rho _S^{\prime } g(t;R^{\prime})c(t;R^{\prime}), \end{aligned}$$

where $\mu _S^{\prime}$ is the molecular dipole moment of the solvent model, $\rho _S^{\prime}$ is the bulk water number density,

20 $$\begin{aligned} g(t;r)=\left( 4\pi \rho _S^{\prime}r^2\Delta r \right) ^{-1} N(t,r;\Delta r), \end{aligned}$$

is the ion–solvent radial distribution function at time $t$ and

21 $$\begin{aligned} c(t;r)= [N(t,r;\Delta r)]^{-1} M(t,r;\Delta r) \end{aligned}$$

is the ion–solvent dipole orientational correlation function at time $t$. Here, $N(t,r;\Delta r)$ is the number of water molecules $j$ for which $r-\Delta r/2 < r_j(t) \le r+\Delta r/2$ ($\mathbf r _j$ denoting the (minimum-image) vector connecting the ion to the oxygen atom of water molecule $j$ and $\Delta r$ the bin width) and $M(t,r;\Delta r)$ is defined as

22 $$\begin{aligned} M(t,r;\Delta r)=\sum _{j,r-\Delta r/2<r_j\le r+\Delta r/2}r_j^{-1}(t)\mathbf r _j(t)\cdot \mathbf {e}_j {(t)}, \end{aligned}$$

$\mathbf e _j$ being a unit vector along the dipole moment of molecule $j$.

The time average of $P(t; R^{\prime}, \mathbf{x})$, evaluated at time $t^{\prime}$, is the average over the sampled trajectory of length $t^{\prime}$,

23 $$\begin{aligned} \langle P(t^{\prime}; R^{\prime}, \mathbf{x})\rangle = {t^{\prime}}^{-1} \int _{0}^{t^{\prime}} \hbox {d}t \, P(t; R^{\prime}, \mathbf{x}), \end{aligned}$$

and is required to be equal to the target value $P_{\rm {tar}}(R^{\prime})$. This can be enforced by application of a corresponding restraining potential, allowing e.g., harmonic deviations according to a force constant $k$ from the target value,

24 $$\begin{aligned} V^{\rm {restr}} ( \langle P(t^{\prime}; R^{\prime}, \mathbf{x}) \rangle ; P_{\rm {tar}}(R^{\prime}), k ) = \frac{1}{2} k \left[ \langle P(t; R^{\prime}, \mathbf{x}) \rangle - P_{\rm {tar}}(R^{\prime}) \right] ^2. \end{aligned}$$

The choice of the target values $P_{\rm {tar}}(R^{\prime})$ is discussed in Sect. 2.4. In practice, the restraint given by Eq. 24 is applied for discrete values $R^{\prime}\in [R_1^{\rm {restr}}; R_2^{\rm {restr}}]$, i.e., there is a number $N_{P}$ of polarization restraints centered at distances $R_n^{\prime}$, where $R_n^{\prime}=R_1^{\rm {restr}}+ (n+0.5) \Delta R_P$, with $n=0,1$,…,$\frac{ R_2^{\rm {restr}} - R_1^{\rm {restr}}}{\Delta R_P}-1$. Therefore, in the following, the notation $R_n^{\prime}\doteq R^{\prime}$ is used.

Since the plain time average of the polarization, as given by Eq. 23 is not suitable to derive restraint forces by differentiating Eq. 24, $\langle P(t^{\prime}; R_n^{\prime}, \mathbf{x}) \rangle$ is estimated in terms of a decay time $\tau$, such that the time average of the polarization sampled at $R_n^{\prime}$ at time $t^{\prime}$ becomes

25 $$\begin{aligned} \overline{ P(t^{\prime}; \tau , R_n^{\prime}, \mathbf{x}) } = \left\{ \tau \left( 1-\exp \left[ -\tau ^{-1} t^{\prime}\right] \right) \right\} ^{-1} \int _{0}^{t^{\prime}} \hbox {d}t \exp \left[ -\tau ^{-1} (t^{\prime}-t) \right] P(t; R_n^{\prime}, \mathbf{x}). \end{aligned}$$

Using the time relaxation formalism of Eq. 25, the restraining potential of Eq. 24 is formally rewritten as

26 $$\begin{aligned} V^{\rm {restr}}( \overline{ P(t; \tau , R_n^{\prime}, \mathbf{x}) } ; P_{\rm {tar}}(R_n^{\prime}), k ) = \frac{1}{2} k \left[ \overline{ P(t; \tau , R_n^{\prime}, \mathbf{x}) } - P_{\rm {tar}}(R_n^{\prime}) \right] ^2, \end{aligned}$$

and if the decay time is much smaller than the simulation length, i.e., $\tau \ll t$, the time average $\overline{ P(t; \tau , R_n^{\prime}, \mathbf{x})}$ given by Eq. 25 can be written in terms of discrete integration time steps $\Delta t$ as [141]

27 $$\begin{aligned} \overline{ P(t; \tau , R_n^{\prime}, \mathbf{x}) } = \left( 1- \exp \left[ -\tau ^{-1} \Delta t\right] \right) P(t; R_n^{\prime}, \mathbf{x}) + \exp \left[ - \tau ^{-1} \Delta t\right] \overline{ P(t-\Delta t; \tau , R_n^{\prime}, \mathbf{x}) } . \end{aligned}$$

The remarks made about Eq. 12 in Sect. 2.2 anologously apply to Eq. 27. One can calculate the restraint forces $\mathbf{F}^{\rm {restr}}_I(t; \mathbf{x})$ acting on the ion and $\mathbf{F}^{\rm {restr}}_i(t; \mathbf{x})$ acting on solvent atom $i$,

28 $$\begin{aligned} \mathbf{F}^{\rm {restr}}_I(t; \mathbf{x}) = - \sum _{i=1}^{N_sN_{s,a}} \mathbf{F}^{\rm {restr}}_i(t; \mathbf{x}) \end{aligned}$$

by differentiating Eq. 26. The force $\mathbf{F}^{\rm {restr}}_I(t; \mathbf{x})$ is thus

29 $$\begin{aligned} \mathbf{F}^{\rm {restr}}_I(t; \mathbf{x})&= - \frac{\partial V^{\rm {restr}} ( \overline{ P(t; \tau , R_n^{\prime}, \mathbf{x})} ; P_{\rm {tar}}(R_n^{\prime}), k ) }{ \partial \mathbf{r}_I} \nonumber \\&= - k \left[ \overline{ P(t; \tau , R_n^{\prime}, \mathbf{x}) } - P_{\rm {tar}}(R_n^{\prime}) \right] \left( \frac{ \partial \overline{ P(t; \tau , R_n^{\prime}, \mathbf{x}) } }{ \partial \mathbf{r}_I} - \frac{ \partial P_{\rm {tar}}(R_n^{\prime}) }{ \partial \mathbf{r}_I} \right) . \end{aligned}$$

The derivative of the Born polarization $P_{\rm {tar}}(R_n^{\prime})$ with respect to the ion position, which is needed in Eq. 29 is here obtained from rewriting $P_{\rm {tar}}(R_n^{\prime})$ in terms of its continuous (rather than binned) analog using the magnitude $r_{Io(i)}$ of the (minimum-image) vector $\mathbf{r}_{Io(i)}$ pointing from the ion to the oxygen atom of molecule $i$, i.e., (see Eq. 18)

30 $$\begin{aligned} P_{\rm {tar}}(r_{Ii}) = \frac{1}{4 \pi r_{Ii}^2} q_I \left( 1- \frac{1}{\epsilon _S^{\prime}}\right) , \end{aligned}$$

so that

31 $$\begin{aligned} \frac{ \partial P_{\rm {tar}}(r_{Ii}) }{ \partial \mathbf{r}_I} = 2 P_{\rm {tar}}(r_{Ii}) r_{Ii}^{-2} \mathbf{r}_{Ii}, \end{aligned}$$

where the abbreviations $\mathbf{r}_{Ii} \doteq \mathbf{r}_{Io(i)}$ and $r_{Ii} \doteq r_{Io(i)}$ were introduced to simplify the notation. It should be noted that the corresponding force term $k \left[ \overline{ P(t; \tau , R_n^{\prime}, \mathbf{x}) } - P_{\rm {tar}}(R_n^{\prime}) \right] \frac{ \partial P_{\rm {tar}}(R_n^{\prime})}{ \partial \mathbf{r}_I}$ in Eq. 29 is vanishing for ion–hydrogen atom interactions, because it is the oxygen atom positions that determine the bin $R_n^{\prime}$ the water molecule belongs to during the simulation (therefore, $R_n^{\prime}$ and $r_{Ii}$ can be used interchangibly in $P_{\rm {tar}}(r_{Ii})$ in Eq. 31). Issues concerning the force due to $\frac{ \partial P_{\rm {tar}}(R_n^{\prime}) }{ \partial \mathbf{r}_I}$ in Eq. 29 are discussed in the "Appendix". In practice, the force due to Eq. 31 will not be applied during the simulation. The reason is that the restraint potential is active in an open system, i.e., within a subsystem of the computational box that does not have a constraint on the number of particles it contains. As a consequence, inclusion of the extra force would contribute to lowering the restraint energy by removing water molecules from the binned region. This undesired behavior could be remedied by using an additional restraint on the number density of particles contained within the binned region, which would be exactly opposite to the force due to Eq. 31. Hence, the extra force due to the position dependence of the target value is omitted.

Using

32 $$\begin{aligned} \mu \frac{\partial r_{Ii}^{-1} \mathbf{r}_{Ii} \cdot \mathbf{e}_i }{\partial \mathbf{r}_I} = \mu \left[ -r_{Ii}^{-1} \mathbf e _i + r_{Ii}^{-3} \mathbf{r}_{Ii} (\mathbf{r}_{Ii} \cdot \mathbf e _i) \right] = -r_{Ii}^{-1} \varvec{\mu }_i + r_{Ii}^{-3} \mathbf{r}_{Ii} (\mathbf{r}_{Ii} \cdot \varvec{\mu }_i) \end{aligned}$$

and Eqs. 19, 21 and 22, one can write

33 $$\begin{aligned} \frac{\partial P(t; \tau , R_n^{\prime}, \mathbf{x}) }{\partial \mathbf{r}_I}= \rho _S^{\prime } g(t; R_n^{\prime}) [N_s(t,R_n^{\prime})]^{-1} \sum _{i=1}^{N_s(t,R_n^{\prime})} \left[ -r_{Ii}^{-1} \varvec{\mu }_i + r_{Ii}^{-3} \mathbf{r}_{Ii} (\mathbf{r}_{Ii} \cdot \varvec{\mu }_i) \right] , \end{aligned}$$

where $N_s(t,R_n^{\prime})$ is the number of water molecules whose oxygen atom is at time $t$ in the bin of width $\Delta R_P$ centered at a distance $R_n^{\prime}$ from the ion and $\varvec{\mu }_i= \mu \mathbf{e}_i$ is the dipole moment vector of molecule $i$. Using Eqs. 27 and 33, one finds

34 $$\begin{aligned} \frac{ \partial \overline{ P(t; \tau , R_n^{\prime}, \mathbf{x}) } }{ \partial \mathbf{r}_I}&= \left( 1- \exp \left[ -\tau ^{-1} \Delta t\right] \right) \rho _S^{\prime } g(t; R_n^{\prime}) [N_s(t,R_n^{\prime})]^{-1} \nonumber \\& \quad \cdot \sum _{i=1}^{N_s(t,R_n^{\prime})} \left[ -r_{Ii}^{-1} \varvec{\mu }_i + r_{Ii}^{-3} \mathbf{r}_{Ii} (\mathbf{r}_{Ii} \cdot \varvec{\mu }_i) \right] \\& \quad + \exp \left[ - \tau ^{-1} \Delta t \right] \frac{\partial \overline{ P(t -\Delta t; \tau , R_n^{\prime}, \mathbf{x}) } }{\partial \mathbf{r}_I}, \quad \nonumber \end{aligned}$$

where the second term on the right-hand side vanishes, i.e., inserting Eqs. 31 and 34 in Eq. 29 one obtains

35 $$\begin{aligned} \mathbf{F}^{\rm {restr}}_I(t; \mathbf{x})&= - k \left[ \overline{ P(t; \tau , R_n^{\prime}, \mathbf{x})} - P_{\rm {tar}}(R_n^{\prime})\right] \left\{ \left( 1- \exp \left[ -\tau ^{-1} \Delta t\right] \right) \right. \nonumber \\& \quad \left. \cdot \rho _S^{\prime } g(t; R_n^{\prime}) [N_s(t,R_n^{\prime})]^{-1} \sum _{i=1}^{N_s(t,R_n^{\prime})} \left[ -r_{Ii}^{-1} \varvec{\mu }_i + r_{Ii}^{-3} \mathbf{r}_{Ii} (\mathbf{r}_{Ii} \cdot \varvec{\mu }_i)\right] \right. \\& \quad \left. - \sum _{i=1}^{N_s(t,R_n^{\prime})} 2 P_{\rm {tar}}(r_{Ii}) r_{Ii}^{-2} \mathbf{r}_{Ii} \right\} . \nonumber \end{aligned}$$

Using Eq. 20, one can simplify Eq. 35 as

36 $$\begin{aligned} \mathbf{F}^{\rm {restr}}_I(t; \mathbf{x})&= - k \left[ \overline{ P(t; \tau , R_n^{\prime}, \mathbf{x})} - P_{\rm {tar}}(R_n^{\prime})\right] \left\{ \left( 1- \exp \left[ -\tau ^{-1} \Delta t\right] \right) \right. \nonumber \\&\left. \cdot (4 \pi {R_n^{\prime}}^2 \Delta R_P)^{-1} \sum _{i=1}^{N_s(t,R_n^{\prime})} \left[ -r_{Ii}^{-1} \varvec{\mu }_i + r_{Ii}^{-3} (\mathbf{r}_{Ii} \cdot \varvec{\mu }_i) \mathbf{r}_{Ii} \right] \right. \\&\left. - \sum _{i=1}^{N_s(t,R_n^{\prime})} 2 P_{\rm {tar}}(r_{Ii}) r_{Ii}^{-2} \mathbf{r}_{Ii} \right\} .\nonumber \end{aligned}$$

For the oxygen and hydrogen coordinate vectors, one gets, similar to Eq. 32,

37 $$\begin{aligned} \frac{\partial r_{Ii}^{-1} \mathbf{r}_{Ii} \cdot \varvec{\mu }_i }{\partial \mathbf{r}_{o(i)}} = r_{Ii}^{-1} \left( \varvec{\mu }_i + \mathbf{r}_{Ii} q_{o(i)} \right) - r_{Ii}^{-3} \mathbf{r}_{Ii} \left( \mathbf{r}_{Ii} \cdot \varvec{\mu }_i \right) \end{aligned}$$

and

38 $$\begin{aligned} \frac{\partial r_{Ii}^{-1} \mathbf{r}_{Ii} \cdot \varvec{\mu }_i }{\partial \mathbf{r}_{h(i)}} = r_{Ii}^{-1} \mathbf{r}_{Ii} q_{h(i)}, \end{aligned}$$

with $h(i)$ denoting a hydrogen atom of molecule $i$. The forces on the solvent oxygen atoms within the bin corresponding to $R_n^{\prime}$ are hence

39 $$\begin{aligned} \mathbf{F}^{\rm {restr}}_{o(i)}(t; \mathbf{x})&= - k \left[ \overline{ P(t; \tau , R_n^{\prime}, \mathbf{x})} - P_{\rm {tar}}(R_n^{\prime})\right] \left\{ \left( 1- \exp \left[ -\tau ^{-1} \Delta t\right] \right) \right. \nonumber \\& \quad \cdot (4 \pi {R_n^{\prime}}^2 \Delta R_P)^{-1} \left[ r_{Ii}^{-1} \varvec{\mu }_i + r_{Ii}^{-1} \mathbf{r}_{Ii} q_{o(i)} - r_{Ii}^{-3} (\mathbf{r}_{Ii} \cdot \varvec{\mu }_i) \mathbf{r}_{Ii} \right] \\& \quad \left. + 2 P_{\rm {tar}}(r_{Ii}) r_{Ii}^{-2} \mathbf{r}_{Ii}\right\} , \nonumber \end{aligned}$$

with $o(i)$ denoting the oxygen atom of molecule $i$. It is emphasized once more that the last term in this force as well as the corresponding term in Eq. 36 is excluded to avoid the need for an additional restraint on the water number density. The corresponding forces on the hydrogen atoms belonging to a water molecule within the bin corresponding to $R_n^{\prime}$ are

40 $$\begin{aligned} \mathbf{F}^{\rm {restr}}_{h(i)}(t; \mathbf{x})&= - k \left[ \overline{ P(t; \tau , R_n^{\prime}, \mathbf{x})} - P_{\rm {tar}}(R_n^{\prime})\right] \left( 1- \exp \left[ -\tau ^{-1} \Delta t\right] \right) \nonumber \\& \quad \cdot (4 \pi {R_n^{\prime}}^2 \Delta R_P)^{-1} r_{Ii}^{-1} \mathbf{r}_{Ii} q_{h(i)}. \end{aligned}$$

In summary, application of the polarization restraint to the long-range regime of the solvent polarization around a charged particle allows to estimate the magnitude of local cutoff-induced artifacts in the polarization in terms of the electrostatic potential sampled at the ion site. Here, “local” means directly at the cutoff distance. These artifacts, which do not occur in continuum-electrostatics representations of the solvent around an ion, [148, 149] and are hence not accounted for by previously proposed continuum-electrostatics-based correction schemes to ion charging free energies [63] can now be quantified and corrected on-the-fly.

Polarization artifacts inside the cutoff sphere of the ion and at the cutoff distance can in principle be ameliorated by increasing the cutoff distance. However, this does (1) not fully eliminate the cutoff artifacts [63] and (2) comes with a significant increase in computation time spent on additional non-bonded solvent–solvent interactions. The latter interactions do not have to be calculated in the case of a polarization restraint (data not shown), even if its range of action extends beyond the cutoff sphere of the ion.

### Restraint targets and generalization to non-spherically symmetric systems

Since the systems considered so far obey spherical symmetry (single ion in water), the target values for the electrostatic potential $\phi _{\rm {tar}}$ and for the polarization $P_{\rm {tar}}(R)$ are constant throughout the simulation. Moreover, these target values are readily available.

$\phi _{\rm {tar}}$ can be determined by adding an electrostatic potential correction term $\tilde{{\phi }}_{ cor }$ to the “raw” electrostatic potential $\phi$ sampled at the ion site in an unrestrained simulation,

41 $$\begin{aligned} \phi _{\rm {tar}} = {\phi } + \tilde{\phi }_{ cor }, \end{aligned}$$

where $\tilde{\phi }_{ cor }$ is given by a subset $\Delta \tilde{A}_{ cor }$ of corresponding well-established correction terms $\Delta A_{ cor }$ for the solvation free energies of monoatomic ions, [63, 64]

42 $$\begin{aligned} \tilde{\phi }_{ cor } = 2 q_I^{-1} \Delta \tilde{A}_{ cor }. \end{aligned}$$

The effect of application of the restraint on the charging free energy of a sodium ion is illustrated in Fig. 1b. For example, if the charging free energy of the ion is calculated via integrating the solvent-generated electrostatic potential at the ion site along discrete charge states varying from zero to full charge, it is clear that the correction $\Delta \tilde{A}_{ cor }$ to the charging free energy will be accounted for if each charge state is simulated with an electrostatic potential restraint to the corresponding corrected electrostatic potential.

Note that in the present case (simulations under periodic boundary conditions), the correction term in Eq. 42 must exclusively account for artifacts that can be captured by a force modification in a periodic system, i.e., $\Delta \tilde{A}_{ cor }$ in Eq. 42 is a solvation free energy correction term for artifacts occurring within the cutoff sphere around an ion simulated with an electrostatic interaction function with cutoff truncation,

43 $$\begin{aligned} \Delta \tilde{A}_{ cor }=\Delta A_{A_2} + \Delta A_{B}, \end{aligned}$$

where $\Delta A_{A_2}$ and $\Delta A_B$ are explained in Sect. 1 and defined in more detail in Refs. [63, 64]. Note that in the latter references, the free energy is denoted with the symbol $G$ instead of $A$. It would not make sense to e.g., include a Born-like correction for the omitted solvent beyond the cutoff sphere in the case of an electrostatic interaction function involving cutoff truncation [63, 64, 138] ($\Delta A_{A_1}$; Sect. 1) or to include a finite-size correction [35, 63, 64, 67] for artificial periodicity in the case of a lattice-sum electrostatic interaction function ($\Delta A_{B}$; Sect. 1). This is because it is not physical to remedy the associated artifacts within a cutoff sphere in the former case or within a box of finite dimensions in the latter case.

The target polarization $P_{\rm {tar}}$ is the polarization around a spherical ion of charge $q_I$ embedded in a macroscopic homogeneous dielectric medium of relative permittivity as appropriate for the employed solvent model, i.e., the Born polarization [150] (Eq. 18).

For oligoatomic ions, these target values have to be determined numerically, e.g., with a finite-difference solver of the Poisson equation. $\phi _{\rm {tar}}$ is the electrostatic potential generated at the ion site by the solvent in a macroscopic nonperiodic system with Coulombic electrostatic interactions, and $P_{\rm {tar}}$ is the underlying solvent polarization, which, for a nonspherical ion, has to be evaluated on a three-dimensional grid around the ion. In addition, for flexible ions, these target values have to be determined numerically on-the-fly during the simulation. This may be done with a Poisson-Boltzmann equation solver [151, 152]. For the sake of computational efficiency, the adequacy and applicability of empirical solutions provided by generalized Born models [153, 154] may be investigated. Especially for systems far more complex than ions in solution, such as solvated biomolecules, which involve larger sizes and a larger number of particles, computationally efficient methods will have to be used to obtain target values for the polarization and/or electrostatic potential. Imagine, for example, the case of a solvated lipid bilayer. Cutoff-truncation schemes are known to introduce artifacts in the simulated properties of these systems [118, 119, 126, 155–157]. A polarization restraint to e.g., the numerically determined ideal (i.e., corresponding to a macroscopic system with Coulombic electrostatic interactions) headgroup polarization could eliminate artifacts in headgroup orientation due to cutoff truncation. Furthermore, a benchmarking against alternative long-range electrostatic treatments would be required. This will, however, be the scope of future work.

The requirement for a user-input target electrostatic potential in the electrostatic potential restraint or target polarization in the polarization restraint and the current limitation to a single ion may be considered disadvantages of the presented approaches. However, the target values are readily available, which is why the authors of the present study think that the methods serve as a useful starting point for further investigations addressing the on-the-fly elimination of electrostatic artifacts in more complex systems.

## Computational details

### Molecular dynamics simulation settings

All MD simulations were performed with a modified version of the GROMOS11 program [158]. All simulations were carried out under periodic boundary conditions (PBC) based on cubic computational boxes containing one sodium ion and 2,142 water molecules. The sodium ion was described according to set L of Ref. [65], and water was described according to the three-site SPC model [159]. The equations of motion were integrated using the leap-frog scheme [160] with a timestep of 2 fs. The rigidity of the water molecules was enforced by application of the SHAKE algorithm [161] with a relative geometric tolerance of $10^{-4}$. The center of mass translation of the computational box was removed every 2 ps. The temperature was maintained at 300 K by weak coupling to a heat bath [162] using a coupling time of 0.1 ps. The box volume was kept constant at $65.94\, \hbox {nm}^3$, which, for the given particle number, corresponds to the equilibrated density of the SPC water model at a temperature of 300 K and a pressure of 1 atm. Electrostatic interactions were either calculated using molecule-based cutoff truncation with a Barker-Watts reaction-field correction [48] (BM; Eq. 6), using molecule-based cutoff truncation without such a correction (CM; Eq. 5) or using atom-based cutoff truncation with such a correction (BA; Eq. 6). The cutoff distance was set to 1.4 nm, and the solvent relative dielectric permittivity entering in the reaction-field terms was set to 66.6, as appropriate for the SPC water model [163]. Van der Waals interactions were calculated using the Lennard-Jones potential, truncated at a distance of 1.4 nm. The pairlist and corresponding interaction energies and forces were updated at each time step. All simulations were equilibrated for 100 ps before a production run of 1 ns length was performed. Coordinates and energies were written to file every 0.1 ps.

The electrostatic potential restraint acts only on solvent molecules within the cutoff sphere of the ion. On the contrary, implementation of the polarization restraint allows a flexible choice of the region of action, and different regions were tested, namely shells extending in ranges 1.0–1.4, 1.0–1.9, 1.1–1.9 and 1.2–1.9 nm from the ion. In all cases, the bin width was set to 0.02 nm. The electrostatic potential and polarization restraints were applied with a decay time $\tau =5$ ps. For the former, the force constants were set to $250.0\, \hbox {kJ}^{-1}\;\hbox {mol}\; e^2$ and for the latter, they were set to $0.75\times 10^7\, \hbox {kJ}\;\hbox {mol}^{-1}\; e^{-2} \;\hbox {nm}^4$ for all simulations except for the case of the BM scheme with application of the restraint in a range of 1.0–1.4 nm from the ion, where it was set to $0.5\times 10^7\, \hbox {kJ}\;\hbox {mol}^{-1}\; e^{-2} \;\hbox {nm}^4$ due to failure of the SHAKE algorithm in the case of a higher force constant.

For the present simulations employing the BM, CM and BA schemes for the treatment of electrostatic interactions, the target electrostatic potentials $\phi _{\rm {tar}}$ (Eq. 41) were set to $-710.86$, $-782.42$ and $-707.71\,\hbox {kJ}\; \hbox {mol}^{-1}\; e^{-1}$, respectively, based on $\phi =\langle \phi ({\mathbf {r}}_I) \rangle$ from the corresponding unrestrained simulations (Table 1) along with $q_I=1.0~e$ and $\Delta \tilde{A}_{ cor }=-26.57\, \hbox {kJ}\;\hbox {mol}^{-1}$ for the schemes with reaction-field correction (BM, BA) or $\Delta \tilde{A}_{ cor }=14.90\, \hbox {kJ}\;\hbox {mol}^{-1}$ for the CM scheme (Eq. 42). The correction terms $\Delta \tilde{A}_{ cor }$ are based on [63, 64] $\Delta A_{A_2}=-24.70$ or $15.46\,\hbox {kJ}\;\hbox {mol}^{-1}$ and $\Delta A_{B}=-1.87$ or $-0.55\, \hbox {kJ}\;\hbox {mol}^{-1}$ for the schemes with reaction-field correction (BM, BA) or the CM scheme, respectively. They were calculated from the fitted functions presented in Ref. [64] along with parameters $\epsilon _{BW}=66.6,$ $R_C=1.4$ nm, $L=4.04$ nm and an effective radius [65] $R_I=0.168$ nm for the $\hbox {Na}^+$ ion. The Born polarization (Eq. 18) was calculated with $\epsilon _S^{\prime}=66.6$, as appropriate for the SPC water model [163].

**Table 1:** Average electrostatic potential $\langle \phi ({\mathbf {r}}_I) \rangle$ and associated root-mean-square fluctuation (rmsf) at the sodium ion site, average physical potential energy per water atom in the restraint region $\langle \tilde{u}_{s}\rangle$ and associated rmsf, as well as total average restraint energy $\langle V^{\rm {restr}}\rangle$ and average restraint energy per water atom in the restraint region $\langle v^{\rm {restr}}\rangle$ monitored during 1 ns simulation of a hydrated sodium ion without (“unres.”) and with (“res.”) application of an electrostatic potential restraint (Eq. 11) to the target value $\phi _{\rm {tar}}$, obtained from Eq. 41. The reported electrostatic potentials $\langle \phi ({\mathbf {r}}_I) \rangle$ and mean potential energies $\langle \tilde{u}_{s}\rangle$ were calculated with the same electrostatic interaction function as used for configurational sampling. $\tilde{u}_{s}$ was calculated for the range 0.0–1.4 nm (Eqs. 45 and 47)

Scheme	$\langle \phi ({\mathbf {r}}_I) \rangle$ (rmsf) [$\hbox {kJ}\;\hbox {mol}^{-1}\; e^{-1}$]		$\phi _{\rm {tar}}$ [$\hbox {kJ}\;\hbox {mol}^{-1}\; e^{-1}$]	$\langle \tilde{u}_{s}\rangle$ (rmsf) [$\hbox {kJ}\;\hbox {mol}^{-1}$]		$\langle V^{\rm {restr}}\rangle$ [$\hbox {kJ}\;\hbox {mol}^{-1}$]	$\langle v^{\rm {restr}}\rangle$ [$\hbox {kJ}\;\hbox {mol}^{-1}$]
	Unres.	Res.		Unres.	Res.	Res.	Res.
BM	−657.72 (43.65)	−709.62 (37.48)	−710.86	−27.67 (0.21)	−27.57 (0.21)	108.92	9.7 $\times\,10^{-2}$
CM	−812.22 (45.66)	−782.56 (40.45)	−782.42	−27.95 (0.22)	−27.90 (0.25)	44.09	3.9 $\times\,10^{-2}$
BA	−654.57 (43.04)	−706.82 (37.56)	−707.71	−27.71 (0.32)	−27.63 (0.34)	111.17	1.0 $\times\,10^{-1}$

Some simulations were also performed with other values of $k$ and $\tau$, but the same target electrostatic potentials and polarizations as reported above. Their results are reported in Supplementary Material.

### Characterization of water density, structure and energetics

The sampled solvent configurations were examined in terms of trajectory averages of the ion-dipole radial distribution function (Eq. 20), the ion-dipole orientational correlation function (Eq. 21) and the radial polarization (Eq. 19), along with [159] $\mu _S^{\prime}=0.0473~e\, {\mathrm {nm}}$ and [163] $\epsilon _S^{\prime}=66.6$ as appropriate for the SPC water model. These trajectory averages are in the following denoted as $g(r)$, $c(r)$ and $P(r)$, respectively, i.e.,

44 $$\begin{aligned} g(r)&= \langle g(t;r) \rangle \nonumber \\ c(r)&= \langle c(t;r) \rangle \\ P(r)&= \mu _S^{\prime}\rho _S^{\prime } g(r)c(r).\nonumber \end{aligned}$$

The polarization $P(r)$ was compared to the Born polarization (Eq. 18) with the same value of the relative dielectric solvent permittivity, $\epsilon _S^{\prime}=66.6$. Unless stated otherwise, the bin width $\Delta r$ for the analysis was set to 0.01 nm.

To characterize the impact of altered water density and polarization on the water–water pairwise interaction energy, the mean water–water interaction energy per water atom in a shell region lying within distances $R_{1}$ and $R_{2}>R_1$ from the ion was calculated for the BA scheme as

45 $$\begin{aligned} \tilde{u}_{s}(R_1,R_2) = N_C^{-1} \sum _{i=1}^{N_s} \sum _{j=1}^{N_{s,a}} u_{s}^{X}(j) H(r_{Ij}-R_1) H(R_2-r_{Ij}) \end{aligned}$$

where $$X=\hbox {BA}$$ and

46 $$\begin{aligned} N_C = \sum _{i=1}^{N_s} \sum _{j=1}^{N_{s,a}} H(r_{Ij}-R_1) H(R_2-r_{Ij}) \end{aligned}$$

and for the BM and CM schemes as

47 $$\begin{aligned} \tilde{u}_{s}(R_1,R_2) = N_C^{-1} \sum _{i=1}^{N_s} H(r_{Io(i)}-R_1) H(R_2-r_{Io(i)}) \sum _{j=1}^{N_{s,a}} u_{s}^\mathrm{X}(j), \end{aligned}$$

where $X=\hbox {BM}$ or CM and

48 $$\begin{aligned} N_C = \sum _{i=1}^{N_s} N_{s,a} H(r_{Io(i)}-R_1) H(R_2-r_{Io(i)}). \end{aligned}$$

In Eqs. 45 and 47, the first and second sums run over all $N_s$ water molecules $i$ and all $N_{s,a}$ atoms $j$ in water molecule $i$, respectively, $r_{Ij}$ denotes the (minimum-image) distance of atom $j$ from the ion, $r_{Io(i)}$ denotes the (minimum-image) distance of the oxygen atom of water molecule $i$ from the ion and $u_{s}^{X}(j)$ is the sum of van der Waals and electrostatic interaction energies of atom $j$ with all other atoms (including the ion) in its cutoff sphere. The electrostatic interaction energies entering in $u_{s}^{X}(j)$ are calculated according to scheme $X$. $H(x)$ is the Heaviside function [$H(x)=1$ if $x>0$ and $H(x)=0$ otherwise]. Since they are computationally expensive, Eqs. 45 and 47 were evaluated based on frames extracted every 5 ps only. The quantity $-(3/2)\tilde{u}_{s}+PV$, where $PV=RT$ is the pressure-volume contribution, is equivalent to the heat of vaporization of a water molecule in the given region around the ion.

### Charging free energy calculation

The free-energy change associated with the reversible charging of the sodium ion in water (Sect. 3.1) was calculated via thermodynamic integration (TI), i.e., integration of the solvent-generated electrostatic potential sampled at the ion site along discrete charge states varying from zero to full charge. The integration was done according to the trapezoidal rule. Twelve charge states $q_i$ were used ($0.0, 0.1, 0.2, \ldots , 0.9, 1.0$ $e$ and 0.05 $e$). The system was as described in Sect. 3.1 (one ion and 2,142 water molecules at constant temperature of 300 K and constant volume of $65.94\,\hbox {nm}^3$). Electrostatic interactions were calculated with the BM scheme based on a cutoff distance $R_C$ of 1.4 nm and a relative dielectric permittivity $\epsilon _{RF}$ of 66.6. At each charge state, the system was equilibrated for 100 ps before a simulation of 1 ns length was used for production. The average electrostatic potential at the ion was calculated from frames written to file every 0.1 ps.

The TI was done in two ways: without and with application of an electrostatic potential restraint (Eq. 11). The target electrostatic potential (Eq. 41) for each charge state $q_i$ is now a function of $q_i$, i.e.,

49 $$\begin{aligned} \phi _{\rm {tar}}(q_i) = {\phi }(q_i) + \tilde{\phi }_{ cor }(q_i), \end{aligned}$$

where $\phi (q_i)$ is the solvent-generated electrostatic potential at the ion of charge $q_i$ in an unrestrained simulation and $\tilde{\phi }_{ cor }(q_i)$ is the electrostatic potential correction for charge state $q_i$. The underlying charging free energy correction terms $\Delta A_{A_2}$ and $\Delta A_B$ (Eq. 43) are based on continuum electrostatics, [63, 64] i.e., obey linear response. Therefore,

50 $$\begin{aligned} \tilde{\phi }_{ cor }(q_i) = q_i q_I^{-1} \tilde{\phi }_{ cor } =2 q_i q_I^{-2} \Delta \tilde{A}_{ cor }, \end{aligned}$$

where Eq. 42 was used to obtain the second expression. $\Delta \tilde{A}_{ cor }$ is given by Eq. 43. For the present case, $\Delta \tilde{A}_{ cor }$ and $\tilde{\phi }_{ cor }$ evaluate to $-26.57\,\hbox {kJ}\;\hbox {mol}^{-1}$ and $-53.14\, \hbox {kJ}\;\hbox {mol}^{-1}\; e^{-1}$, respectively (Sect. 3.1). The values $\tilde{\phi }_{ cor }(q_i)$ and the resulting target electrostatic potentials $\phi _{\rm {tar}}(q_i)$ are reported in Table S2 in Supplementary Material.

## Results

### Electrostatic potential restraint

Table 1 reports the average electrostatic potential monitored at the sodium ion site in simulations without and with the electrostatic potential restraint. Clearly, the restraint achieves an altered hydration of the ion according to the desired target electrostatic potential $\phi _{\rm {tar}}$ (Eq. 41), while the root-mean-square fluctuations in the electrostatic potential are only moderately reduced in comparison with the unrestrained simulations, namely by 14.1, 11.4 and 12.7 % for the BM, CM and BA simulation, respectively. The magnitude of the fluctuations can be controlled by changes in the force constant and decay time, as illustrated in Supplementary Material (Table S1). A decrease of $k$ and an increase of $\tau$ cause the restraint to be satisfied less well. Lower values of $k$ in combination with decay times $\tau$ that allow the target values to be satisfied effect slightly higher root-mean-square fluctuations in the target electrostatic potential.

In comparison with the average physical potential energy per water atom in the restraint region (i.e., within the cutoff sphere of the ion), $\langle \tilde{u}_s \rangle$ (Eqs. 45, 47), the average restraint energy per water atom $\langle v^{\rm {restr}}\rangle$ , is extremely small. It amounts to only 0.4, 0.1 and 0.4 % of the magnitude of $\langle \tilde{u}_s \rangle$ for the BM, CM and BA simulations, respectively. For the restrained and unrestrained simulations, $\langle \tilde{u}_s \rangle$ is identical to within the root-mean-square fluctuations. Thus, one may conclude that the electrostatic potential restraint induces little perturbation in the solvent–solvent interactions.

**Fig. 2:**
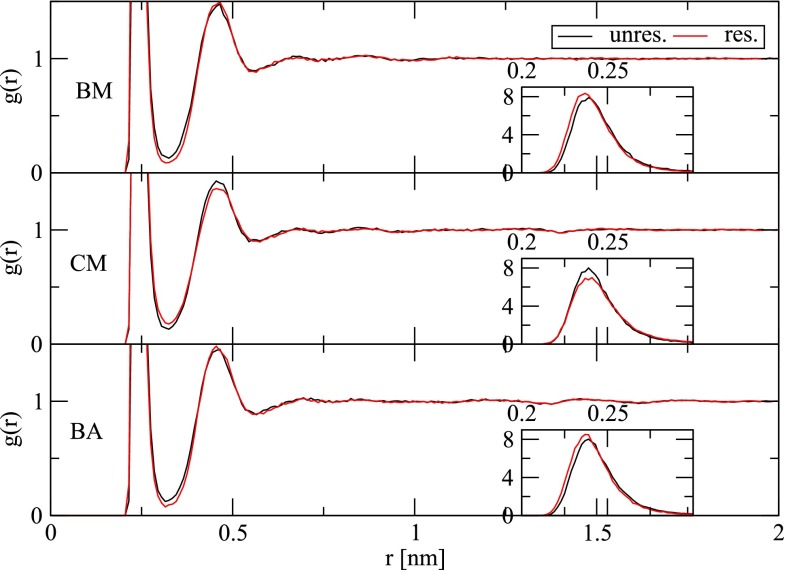
Radial distribution function $g(r)$ (Eq. 44) of water oxygen atoms around the sodium ion for simulations in the absence (“unres.”) or presence (“res.”) of an electrostatic potential restraint (Eq. 11) and involving the BM, CM or BA scheme for the treatment of electrostatic interactions (Sect. 3.1). The inset graphs depict a zoom on the first peak of $g(r)$ evaluated with a finer bin width (0.002 nm) to clearly illustrate the difference in peak heights

**Fig. 3:**
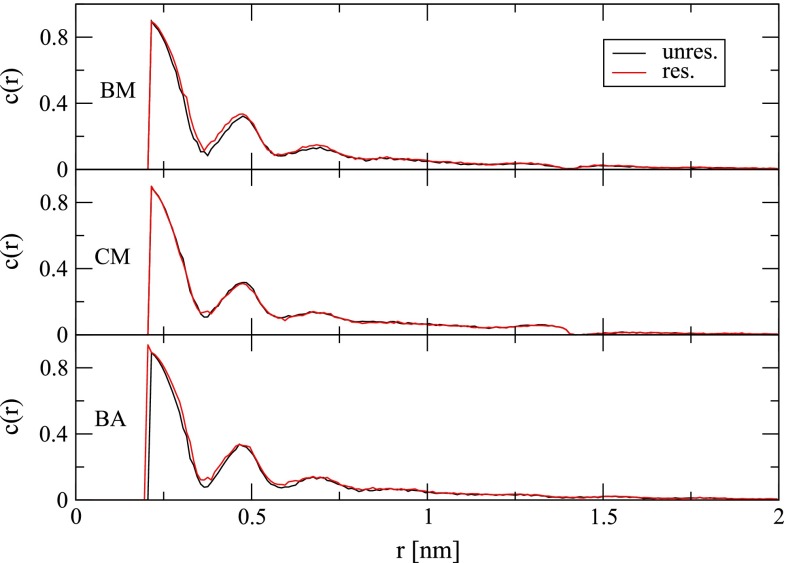
Orientational correlation function $c(r)$ (Eq. 44) of water dipole moment vectors and the vectors connecting corresponding oxygen atoms and the sodium ion site for simulations in the absence (“unres.”) or presence (“res.”) of an electrostatic potential restraint (Eq. 11) and involving the BM, CM or BA scheme for the treatment of electrostatic interactions (Sect. 3.1)

It can be seen from Eq. 17 that the electrostatic potential restraint forces relate to the “normal” electrostatic forces through a scalar factor. The concomitant effect on water density can be seen in Fig. 2. With the BM and BA schemes, the ion is underhydrated in the unrestrained simulations in comparison with the target electrostatic potential $\phi _{\rm {tar}}$ (Table 1). This underhydration is remedied by the restraint through an increased water density around the ion, as evidenced by increased heights of the first peak of the ion–water radial distribution (Fig. 2). In contrast, with the CM scheme, the ion is overhydrated in the unrestrained simulations in comparison with the target electrostatic potential $\phi _{\rm {tar}}$ (Table 1). This overhydration is remedied by the restraint through a reduced water density around the ion, as evidenced by a reduced height of the first peak of the ion–water radial distribution (Fig. 2). Note that the height of the first peak in $g(r)$ from restrained simulations differs between the BM (8.31), CM (6.95) and BA (8.45) simulations. In particular, it is markedly lower for the CM scheme, which is probably due to the strong overpolarization shortly before the cutoff distance caused by the absence of a reaction field. This is illustrated here by the bump in $c(r)$ at distances of 1.25–1.38 nm from the ion (Fig. 3). Besides the ion–water radial distribution function, the electrostatic potential restraint also appears to slightly affect the ion–water dipole orientational correlation function (Fig. 3). Although this is at first glance not expected based on the functional form of the restraint forces, it might be a consequence of the altered water density.

**Fig. 4:**
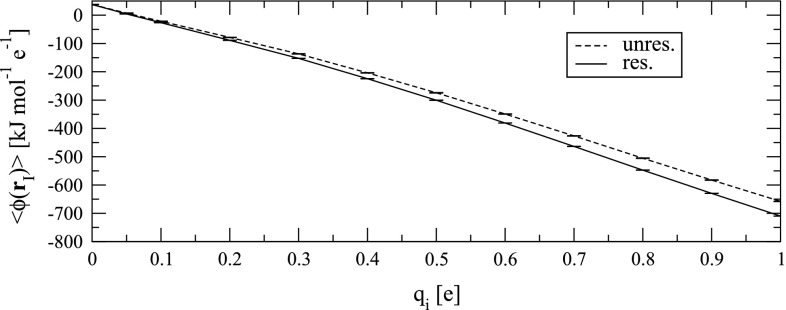
Average electrostatic potential $\langle \phi ({\mathbf {r}}_I) \rangle$ sampled at the site of a hydrated particle with the van der Waals parameters of a sodium ion and a charge $q_i$, monitored during 1 ns simulations without (“unres.”) and with (“res.”) application of an electrostatic potential restraint (Sect. 3.3). *Error bars* illustrate the size of the statistical error calculated via block averaging [19]. The numerical values for $\langle \phi ({\mathbf {r}}_I) \rangle$ are reported in Table S2 in Supplementary Material

The charging free energy of the sodium ion was determined as described in Sect. 3.3. Integration of the TI curve (Fig. 4) leads to $\Delta A_{ chg }=-288.8\pm 0.6\, \hbox {kJ}\;\hbox {mol}^{-1}$ if no electrostatic potential restraint is applied (Fig. 1a). However, if the simulation of each charge state of the ion involves a restraint to a target electrostatic potential $\phi _{\rm {tar}}(q_i)$ appropriate for that charge state (Eq. 49), the resulting charging free energy includes the correction term $\Delta \tilde{A}_{ cor }$ (Eq. 43). Integration of the TI curve (Fig. 4) hence leads to $\Delta A_{ chg }=-315.1\pm 0.3\,\hbox {kJ}\;\hbox {mol}^{-1}$ (Fig. 1b). The small numerical difference in comparison with $\Delta A_{ chg }+\Delta \tilde{A}_{ cor }=(-288.8-26.6)~\mathrm{kJ}\;\mathrm{mol}^{-1} =-315.4\, \hbox {kJ}\;\hbox {mol}^{-1}$ is essentially within the statistical uncertainty. Although the electrostatic potential restraint thus improves the simulated charging free energy (in the sense that the post-simulation correction term to be added becomes smaller in magnitude), the ion is still underhydrated in comparison with the ideal situation. In other words, the ion–water forces during the simulation result in a “raw” hydration free energy ($-315.1\,\hbox {kJ}\;\hbox {mol}^{-1}$) that still has a smaller magnitude than the target value ($-440.9\, \hbox {kJ}\;\hbox {mol}^{-1}$; Fig. 1). The difference is due to corrections not accounted for by the electrostatic potential restraint. Part of it ($\Delta A_{A_1} = -48.9\, \hbox {kJ}\;\hbox {mol}^{-1}$ in the present example) could be reduced by increasing the cutoff distance, which goes, however, with a prohibitive increase in computational expense. The bulk of the remaining part is sizeable and inherent to the nature of the reaction-field correction ($\Delta A_{C_1} = -75.7\, \hbox {kJ}\;\hbox {mol}^{-1}$ in the present example). This may suggest that for ion simulations with an electrostatic potential restraint, the CM scheme (i.e., molecule-based cutoff truncation without reaction-field correction), and as large a cutoff distance as computationally affordable are a worthwile option. Indeed, the remaining unaccounted correction term is of smallest magnitude in this case (Fig. 1b). Yet, since the CM electrostatic interaction function leads to severe cutoff artifacts, [63] additional inclusion of the polarization restraint in the vicinity of the cutoff distance would be highly useful.

The statistical uncertainty in the charging free energy obtained from restrained simulations is smaller than in the unrestrained simulations, which is due to the restraint affecting the magnitude of electrostatic potential fluctuations. In this context, it is important to note that the influence of the restraint on the magnitude of electrostatic potential fluctuations prohibits free-energy calculation methods which rely on cumulant expansions of the electrostatic potential [35, 164] or analytical fitting schemes [165].

### Polarization restraint

**Fig. 5:**
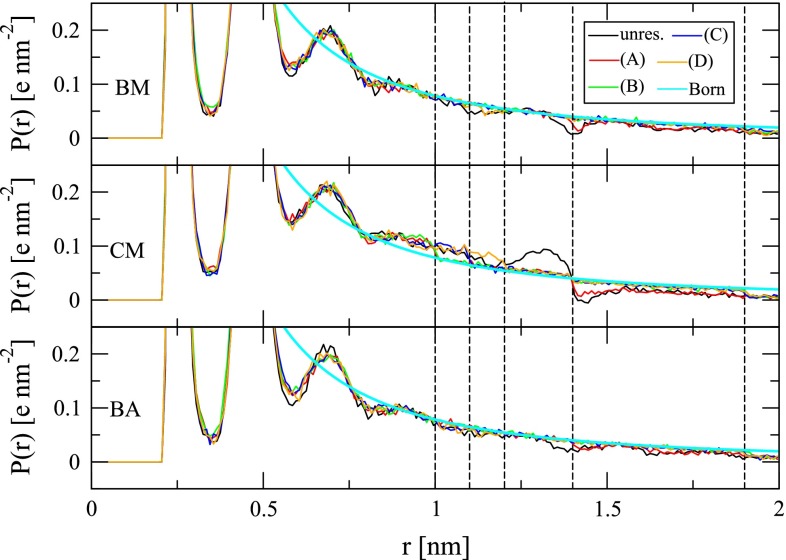
Radial polarization $P(r)$ (Eq. 44) of water around the sodium ion for simulations in the absence (“unres.”) or presence (*A*-*D*) of a polarization restraint (Eq. 26) and involving the BM, CM or BA scheme for the treatment of electrostatic interactions (Sect. 3.1). The *cyan line* depicts the Born polarization (Eq. 18). The *dashed vertical lines* are a guide for the eye and indicate distances of 1.0, 1.1, 1.2, 1.4 and 1.9 nm from the ion. The polarization restraint was applied in spherical shells extending from 1.0 to 1.4 (*A*), 1.0–1.9 (*B*), 1.1–1.9 (*C*) or 1.2–1.9 nm (*D*) around the ion

**Fig. 6:**
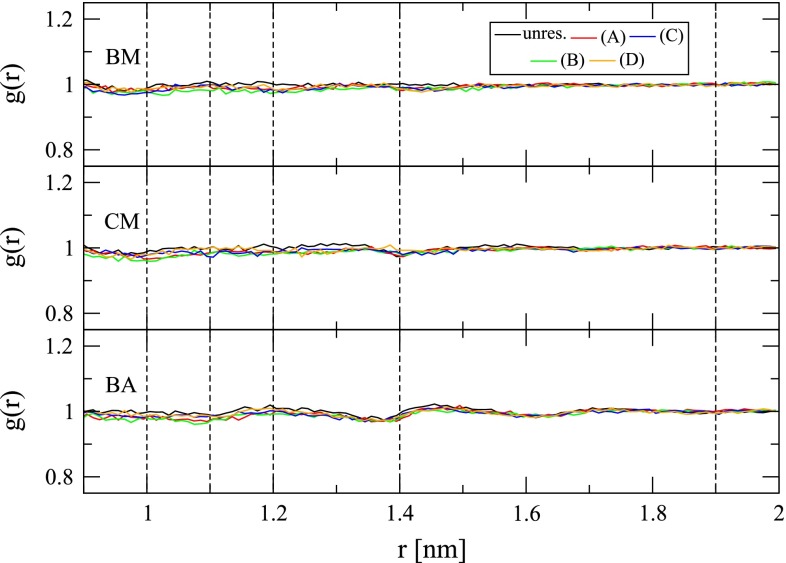
Radial distribution function $g(r)$ (Eq. 44) of water oxygen atoms around the sodium ion for simulations in the absence (“unres.”) or presence (*A*–*D*) of a polarization restraint (Eq. 26) and involving the BM, CM or BA scheme for the treatment of electrostatic interactions (Sect. 3.1). The *dashed vertical lines* are a guide for the eye and indicate distances of 1.0, 1.1, 1.2, 1.4 and 1.9 nm from the ion. The polarization restraint was applied in spherical shells extending from 1.0 to 1.4 (*A*), 1.0–1.9 (*B*), 1.1–1.9 (*C*) or 1.2–1.9 nm (*D*) around the ion

The impact of cutoff-truncated electrostatic interaction functions on the solvent density, orientational correlation and polarization around ionic solutes has been discussed in detail before [63, 65]. Here, only artifacts transpiring in the immediate vicinity of the cutoff distance shall be pointed out again. The radial solvent polarization $P(r)$ exhibits an artificial dip at $R_C$ with the BM and BA schemes. By virtue of atom-based rather than molecule-based cutoff truncation, this dip is less pronounced with the BA scheme (Fig. 5). However, interestingly, artifacts in $g(r)$ are more pronounced with the BA scheme than with the BM scheme (Fig. 6). Obviously, omission of a reaction-field correction has severe effects on the solvent polarization. For the CM scheme, $P(r)$ shows strong overpolarization immediately before and underpolarization immediately after $R_C$. Application of a polarization restraint successfully removes these artifacts (Fig. 5). If the range of action of the polarization restraint is extended beyond the immediate neighborhood of $R_C$, further polarization artifacts can be rectified. This is evident for the CM scheme, where the spurious overpolarization in $P(r)$ was addressed by e.g., applying the restraint in the shell between 1.0 and 1.4 nm from the ion. Since the polarization restraint was implemented such that also water molecules outside the cutoff sphere of the ion can be involved, the underpolarization normally occurring outside the cutoff sphere of the ion can also be corrected, e.g., as done here, up to a distance of 1.9 nm from the ion (Fig. 5). For the system investigated in this study, another advantage of the polarization restraint is that it achieves a long-range polarization closer to the Born polarization than obtained from a simulation with a lattice-sum electrostatic interaction function in a computational box of the same edge length (4.04 nm). This finding is depicted and discussed (along with thermodynamic considerations) in Figure S1 in Supplementary Material.

**Fig. 7:**
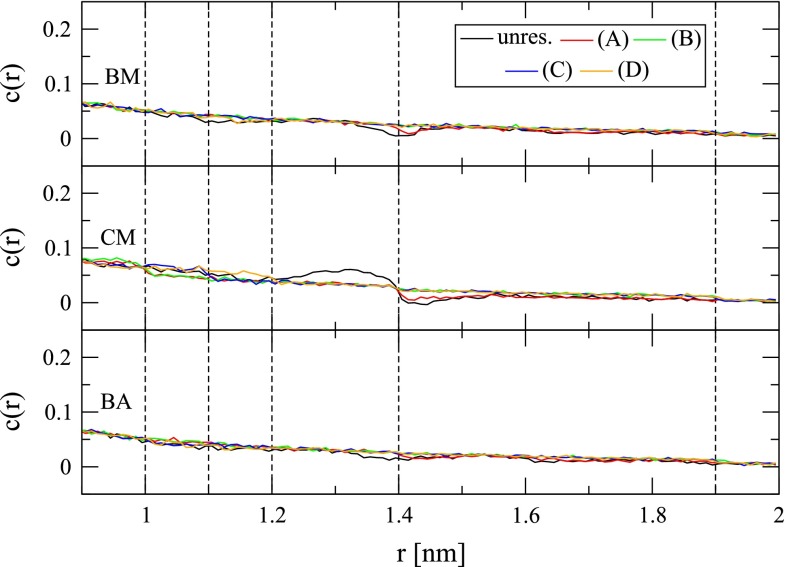
Orientational correlation function $c(r)$ (Eq. 44) of water dipole moment vectors and the vectors connecting corresponding oxygen atoms and the sodium ion site for simulations in the absence (“unres.”) or presence (*A*-*D*) of a polarization restraint (Eq. 26) and involving the BM, CM or BA scheme for the treatment of electrostatic interactions (Sect. 3.1). The *dashed vertical lines* are a guide for the eye and indicate distances of 1.0, 1.1, 1.2, 1.4 and 1.9 nm from the ion. The polarization restraint was applied in spherical shells extending from 1.0–1.4 (*A*), 1.0–1.9 (*B*), 1.1–1.9 (*C*) or 1.2–1.9 nm (*D*) around the ion

Within the region where it was applied, the polarization restraint was not found to affect the ion–water radial distribution function (Fig. 6), while the ion-dipole orientational correlation function reflects the changes already observed in $P(r)$ (Fig. 7). The changes in water molecular orientation are effected by the term in square brackets in the first sum of Eq. 35. This term is the partial derivative of the component of the water molecular dipole moment along the ion-oxygen connecting vector with respect to the position of the ion.

The effect of alternative choices for $\tau$ and $k$ is shown in Figure S2 of the Supplementary Material. A decrease of $k$ and an increase of $\tau$ cause the restraint to be satisfied less well. Obviously, for large values of $\tau$, an increase of $k$ achieves closer agreement with the target polarization.

Table 2 provides information concerning the impact of the polarization restraint on energetic properties. When the range of action of the restraint is restricted to a small part of the cutoff sphere of the ion (restraint range of 1.2–1.9 nm), the ion is better hydrated in the case of the BM scheme by about $2.7\, \hbox {kJ}\;\hbox {mol}^{-1}\; e^{-1}$ because the dip in the solvent polarization is removed. The effect is basically absent in the case of the BA scheme. In the case of the CM scheme, the correction of the overpolarization around 1.2–1.4 nm (restraint range of 1.2–1.9 nm) reduces the electrostatic potential sampled at the ion site by $8.7\,\hbox {kJ}\;\hbox {mol}^{-1}\; e^{-1}$. An increase in the range of action of the restraint, to e.g., 1.0 nm from the ion (restraint range of 1.0–1.9 nm), leads to a reduced hydration of the ion with all three investigated cutoff-truncation schemes in comparison with the unrestrained situation. This is due to a removal of overpolarization within the cutoff sphere. Note that since the ion is only interacting with water molecules within a range of $R_C=1.4$ nm, restraint ranges of 1.0–1.4 and 1.0–1.9 nm give very similar results concerning the electrostatic potential sampled at the ion site.

**Table 2:** Average electrostatic potential $\langle \phi ({\mathbf {r}}_I) \rangle$ and associated root-mean-square fluctuation (rmsf) at a sodium ion site, average physical potential energy per water atom in the restraint region $\langle \tilde{u}_{s}\rangle$ and associated rmsf, as well as total average restraint energy $\langle V^{\rm {restr}}\rangle$ and average restraint energy per water atom in the restraint region $\langle V^{\rm {restr}}\rangle$ monitored during 1 ns simulation of a hydrated sodium ion without (“unres.”) and with application of a polarization restraint (Eq. 26) to the Born polarization (Eq. 30, using $\epsilon _S^{\prime}=66.6$) within the reported ranges of 1.0–1.4, 1.0–1.9, 1.1–1.9 and 1.2–1.9 nm from the ion. $\tilde{u}_{s}$ was calculated for the range 0.0–1.4 nm for unrestrained simulations or for the restraining range in restrained simulations (Eqs. 45 and 47)

Scheme	Res. range (nm)	$\langle \phi ({\mathbf {r}}_I) \rangle$ (rmsf) [$\hbox {kJ}\;\hbox {mol}^{-1}\; e^{-1}]$	$\langle \tilde{u}_{s}\rangle$ (rmsf) [$\hbox {kJ}\;\hbox {mol}^{-1}]$	$\langle V^{\rm {restr}}\rangle$ [$\hbox {kJ}\;\hbox {mol}^{-1}$]	$\langle v^{\rm {restr}}\rangle$ [$\hbox {kJ}\;\hbox {mol}^{-1}$]
BM	Unres.	−657.72 (43.65)	−27.67 (0.21)	–	–
	1.0–1.4	−655.89 (44.90)	−27.19 (0.31)	4,985.8	7.1
	1.0–1.9	−656.43 (44.83)	−27.19 (0.15)	10,895.8	4.6
	1.1–1.9	−660.72 (43.97)	−27.32 (0.17)	9,984.7	4.5
	1.2–1.9	−660.41 (43.85)	−27.41 (0.18)	8,822.9	4.3
CM	Unres.	−812.22 (45.66)	−27.95 (0.22)	–	–
	1.0–1.4	−788.98 (47.58)	−27.28 (0.32)	5,944.1	8.5
	1.0–1.9	−792.51 (47.39)	−27.49 (0.17)	13,284.2	5.6
	1.1–1.9	−798.73 (46.89)	−27.61 (0.17)	11,919.1	5.3
	1.2–1.9	−803.56 (46.58)	−27.69 (0.17)	10,658.6	5.1
BA	Unres.	−654.57 (43.04)	−27.71 (0.32)	–	–
	1.0–1.4	−651.95 (44.30)	−26.97 (0.56)	4,988.1	7.2
	1.0–1.9	−651.78 (45.22)	−27.22 (0.25)	11,093.5	4.7
	1.1–1.9	−654.68 (44.87)	−27.34 (0.25)	9,924.6	4.4
	1.2–1.9	−654.32 (43.97)	−27.44 (0.26)	8,990.5	4.3

Table 2 also reports the root-mean-square fluctuations of the electrostatic potential at the ion site. They are marginally increased upon introduction of the polarization restraint, namely by 0.5–2.9, 2.0–4.2 and 2.2–5.1 % for the BM, CM and BA simulations, respectively. The difference between $\langle \tilde{u}_s \rangle$ (Eqs. 45, 47) from unrestrained and restrained simulations is slightly larger than that in the case of the electrostatic potential restraint. Overall, it is largest when the polarization restraint is applied in a range of 1.0–1.4 nm from the ion (0.48, 0.67 and $0.74\,\hbox {kJ}\;\hbox {mol}^{-1}$ for the BM, CM and BA simulations, respectively), which is likely due to the polarization at very small distances (1.0–1.1 nm) from the ion still presenting minor short-range structural features. As a consequence, restraining to a continuum-like polarization exempt of solvation structure comes at the cost of a greater perturbation of solvent–solvent interactions than is the case when a polarization restraint is applied in the long-range regime of the polarization. This is also reflected by the average restraint energy per water atom. It is larger when the polarization restraint is applied in the range of 1.0–1.4 nm ($\langle v^{\rm {restr}} \rangle =7.1$, 8.5 and $7.2\, \hbox {kJ}\;\hbox {mol}^{-1}$ for the BM, CM and BA schemes, respectively) than for the other investigated restraint regions (on average, $\langle v^{\rm {restr}} \rangle =4.5$, 5.3 or $4.6\, \hbox {kJ}\;\hbox {mol}^{-1}$ for the BM, CM and BA schemes, respectively).

## Conclusion

The use of an effective electrostatic interaction function leads to artifacts in the solvent polarization around an ion. Two possible approaches to correct for such artifacts during the simulation were presented as a possible first step toward simulation protocols exempt of artifacts due to the use of approximate-electrostatics schemes. The force corrections derive from special potential energy terms that restrain (1) the solvent-generated electrostatic potential sampled at a given atom site to a target value involving previously proposed continuum-electrostatics-based corrections for electrostatic artifacts [63, 64] (“electrostatic potential restraint”) or (2) the long-range regime of the solvent polarization around a given atom site to the Born polarization, i.e., the solvent polarization corresponding to the ideal situation of a macroscopic system under nonperiodic boundary conditions and governed by Coulombic electrostatic interactions (“polarization restraint”). Application of the restraints was illustrated for the case of a hydrated sodium ion, simulated with electrostatic interaction functions using molecule-based cutoff truncation with or without a Barker-Watts reaction-field correction, or using atom-based cutoff truncation with a Barker-Watts reaction-field correction. It was seen that the electrostatic potential restraint enforces the target electrostatic potential at the ion site by altering water density while only slightly affecting ion–water orientational correlation or the water–water interactions. The polarization restraint enforces a target dipole moment density in a given distance range from the ion. Thus, predominantly, the ion–water orientational correlation is modified, while the water density remains essentially unaltered.

Obvious limitations of both restraints are the requirement of input target values for either the electrostatic potential or the polarization. Since the system studied here is spherically symmetric, consists of a single solute and was simulated at constant volume, determination of the target values was straightforward. The extension of the presented methodology to the case of multiple solutes is in principle possible. In this sense, the study is a promising step toward the on-the-fly elimination of finite-size and approximate-electrostatic artifacts during atomistic molecular dynamics simulations and a useful starting point for further investigations. It is an alternative to certain established a posteriori corrections for electrostatic artifacts and has the clear advantage of rendering solvent configurational sampling more conform with the ideal situation of a macroscopic nonperiodic system with Coulombic electrostatic interactions. Note in this context that the electrostatic potential restraint was formulated such that it does not act on solvent molecules beyond the cutoff sphere of the ion, whereas the polarization restraint was formulated to also act on solvent molecules outside the cutoff sphere of the ion. The range of action of the former restraint may, however, be trivially extended.

Lastly, it should be emphasized that both restraints can also be used in simulations with a lattice-sum electrostatic interaction function. For the electrostatic potential restraint, the corresponding correction to the electrostatic potential should not be a global finite-size correction (here $\Delta A_B$; $\Delta G_B$ in Refs. [63, 64]), but should only be that portion of the overall periodicity artifacts the water in the simulation box can actually account for. Concerning the alleviation of artificial periodicity artifacts incurred by usage of a lattice-sum electrostatic interaction function, the authors also note very interesting alternative approaches, e.g., orientational averaging of the lattice-sum electrostatic potential [30, 36, 38–40] or combination of the lattice-sum interaction function with other nonperiodic functions, [53, 95–99] as well as the probably most pragmatic approach pertinent to biomacromolecular simulation, inclusion of a screening counterion density.

### Electronic supplementary material

Below is the link to the electronic supplementary material.

Supplementary material 1 (pdf 81 KB)

## Appendix

Here, two points concerning the implementation of the force due to $\frac{ \partial P_{\rm {tar}}(R_n^{\prime}) }{ \partial \mathbf{r}_I}$ in Eq. 29 are discussed. Since $R_n^{\prime}$ denotes the bin in which the oxygen atom of water molecule $i$ resides, the equivalent expression $\frac{ \partial P_{\rm {tar}}(r_{Ii}) }{ \partial \mathbf{r}_I}$ may be used in the following.

First, note that the inclusion of the force due to $\frac{ \partial P_{\rm {tar}}(R_n^{\prime}) }{ \partial \mathbf{r}_I}$ in Eq. 29 can in principle also be implemented in a numerical rather than analytical fashion. It can easily be shown that this extra force (note the sign reversal in comparison with Eq. 29, which denotes a force acting on the ion)

51 $$\begin{aligned} \mathbf{F}_{\hbox{extra},o}(t; \mathbf{x}) = - k \left[ \overline{ P(t; \tau , R_n^{\prime}, \mathbf{x}) } - P_{\rm {tar}}(R_n^{\prime}) \right] \frac{ \partial P_{\rm {tar}}(R_{n}^{\prime}) }{ \partial \mathbf{r}_I} \end{aligned}$$

acting on the oxygen atoms is equivalent to a force due to the restraint energy gradient between successive bins,

52 $$\begin{aligned} \mathbf{F}_{\hbox{extra},o}(t; \mathbf{x})&= - r_{Ii}^{-1} \mathbf{r}_{Ii} \frac{k}{4 \Delta R_P} \left\{ \left[ \overline{ P(t; \tau , R_n^{\prime}, \mathbf{x}) } - P_{\rm {tar}}(R_+^{\prime}) \right] ^2 \right. \nonumber \\& \quad \left. - \left[ \overline{ P(t; \tau , R_n^{\prime}, \mathbf{x}) } - P_{\rm {tar}}(R_-^{\prime}) \right] ^2 \right\} \\&= - r_{Ii}^{-1} \mathbf{r}_{Ii} \frac{k}{4 \Delta R_P} \left\{ \left[ 2 \overline{ P(t; \tau , R_n^{\prime}, \mathbf{x}) } - P_{\rm {tar}}(R_+^{\prime}) - P_{\rm {tar}}(R_-^{\prime}) \right] \right. \nonumber \\& \quad \left. \cdot \left[ P_{\rm {tar}}(R_-^{\prime}) - P_{\rm {tar}}(R_+^{\prime}) \right] \right\} \nonumber \\&= -k \left[ \overline{ P(t; \tau , R_n^{\prime}, \mathbf{x}) } - P_{\rm {tar}}(R_n^{\prime}) \right] \frac{\partial P_{\rm {tar}}(r_{Ii}) }{\partial \mathbf{r}_I }, \quad \nonumber \end{aligned}$$

where $P_{\rm {tar}}(R_+^{\prime})$ and $P_{\rm {tar}}(R_-^{\prime})$ denote the reference Born polarizations in the bins corresponding to $R_+^{\prime}=R_n^{\prime}+\Delta R_P$ and $R_-^{\prime}=R_n^{\prime}-\Delta R_P$, respectively, and the step from the second to the third equality involves the limit $\Delta R_P\rightarrow 0$ along with consequentially identifying

53 $$\begin{aligned} \frac{ P_{\rm {tar}}(R_-^{\prime}) - P_{\rm {tar}}(R_+^{\prime})}{ 2 \Delta R_P} \rightarrow -\frac{\partial P_{\rm {tar}}(R_n^{\prime})}{\partial r} = -\frac{\partial P_{\rm {tar}}(r_{Ii})}{\partial r_{Ii}} = \frac{\partial P_{\rm {tar}}(r_{Ii})}{\partial \mathbf{r}_{I}} \left( \frac{\mathbf{r}_{Ii}}{r_{Ii}} \right) ^{-1} \end{aligned}$$

and

54 $$\begin{aligned} \frac{ P_{\rm {tar}}(R_-^{\prime}) + P_{\rm {tar}}(R_+^{\prime})}{2} \rightarrow P_{\rm {tar}}(R_n^{\prime}) = P_{\rm {tar}}( r_{Ii}). \end{aligned}$$

(The unphysical term $(\mathbf{r}_{Ii})^{-1}$ in Eq. 53 occurs here only formally, because it cancels with $\mathbf{r}_{Ii}$ in the second equality of Eq. 52.)

Second, note that since the restraint potential is active in an open system, i.e., in shells around the ion which water molecules are free to enter and leave, application of the force due to Eq. 31 has an undesired consequence: The restraint energy will be decreased by water molecules leaving those regions of the systems where the restraint is applied. In order to avoid the artificial depletion of water molecules in those regions, an additional restraint on the number density of water, e.g., in the form of a restraint of $g(r)$ (Eq. 20), would have to be used.
