# Supplementary material for: Toward the correction of effective electrostatic forces in explicit-solvent molecular dynamics simulations: restraints on solvent-generated electrostatic potential and solvent polarization
Source: Theor Chem Acc. 2015 Jan 10;134(2):2. doi: 10.1007/s00214-014-1600-8 (PMC4470580; doi:10.1007/s00214-014-1600-8)
Supplement: Supplementary file 1 — Supplementary material 1 (pdf 81 KB) [file 214_2014_1600_MOESM1_ESM.pdf]

Supplementary Material

to

Towards the correction of effective electrostatic forces in explicit-solvent molecular dynamics simulations: Restraints on solvent-generated electrostatic potential and solvent polarization

Maria M. Reif and Chris Oostenbrink

In Section S.I of this document, the long-range solvent polarization around the ion in the system investigated in this study is compared to the polarization obtained from a simulation with a lattice-sum (LS) electrostatic interaction function. See also Section IV.1 of the main article.

Sections S.II and S.III of this document provide supplementary information concerning the impact of a variation of the force constant k and the decay time τ in the restraints discussed in the main article. See also Sections IV.1 and IV.2 of the main article for a short discussion of the results presented here.

Section S.IV of this document provides the numerical values of the thermodynamic integration curves shown in Figure 4 of the main article and additional data pertaining to the underlying simulations.

S.I Comparison of the long-range polarization to results from a simulation with lattice-sum electrostatic interactions

Figure S1 shows the radial polarization of water around the sodium ion for simulations with truncated electrostatic interactions (without and with polarization restraint) and for a LS simulation (without polarization restraint). Since it does not truncate electrostatic interactions, a LS electrostatic interaction function does not introduce cutoff artifacts in the solvent polarization around charged solute molecules. This is clearly visible from Figure S1. However, due to the presence of periodic solute copies, an underpolarization of the solvent is observed throughout the box. Such an underpolarization can be removed by a polarization restraint. For instance, the underpolarization beyond the cutoff distance that is observed in simulations with truncated electrostatic interactions can be corrected (Figure S1).

The solvent underpolarization in LS simulations was discussed before and it was illustrated that the underpolarization artifact, as well as the corresponding effect on the ion hydration free energy, decreases upon increasing the edge length of the computational box.[63] In the present simulations, the box-edge length is 4.04 nm (Section III.1 of the main article). In the case of the LS simulation, one can clearly see the underpolarization of water in comparison to the ideal Born polarization. This artifact is present although a fairly large computational box is used to accommodate the monoatomic ion. Note that the corresponding thermodynamic artifact in the hydration free energy is sizeable. It can be estimated based on a continuum electrostatics model,[18,67] and, in the system considered here, evaluates to 47.9 kJ·mol^{-1}. In other words, the underpolarization of the solvent due to the periodicity inherent in the LS simulations causes the sodium ion (of radius 0.168 nm in a cubic box of edge length 4.04 nm) to be underhydrated by 47.9 kJ·mol^{-1}. For the sake of completeness it should be mentioned that this is not the only artifact affecting hydration free energies in a LS simulation. A problem related to the summation over charge contributions to the electrostatic potential leads to another error in the hydration free energy of, in the present case, 77.5 kJ·mol^{-1}. In total, the hydration free energy of the sodium ion with a LS scheme amounts to -314.5 kJ·mol^{-1}. This is of similar magnitude as the hydration free energies obtained from cutoff-truncation schemes (Figure 1 in the main article) and is (accidentally) remarkably close to the raw charging free energy obtained from a truncation scheme upon inclusion of the electrostatic potential restraint (-315.4 kJ·mol^{-1}; Section IV.1 in the main article).

At first glance, applying a polarization restraint to a LS simulation seems to be a good solution to the problem of underpolarization. However, as all the periodic solvent copies contribute to the electrostatic potential at the ion, another artifact would be created if a polarization restraint was applied because solvent molecules in periodic box copies still would have a spurious polarization due to artificial periodicity, which cannot be corrected for by the application of the restraints described here.

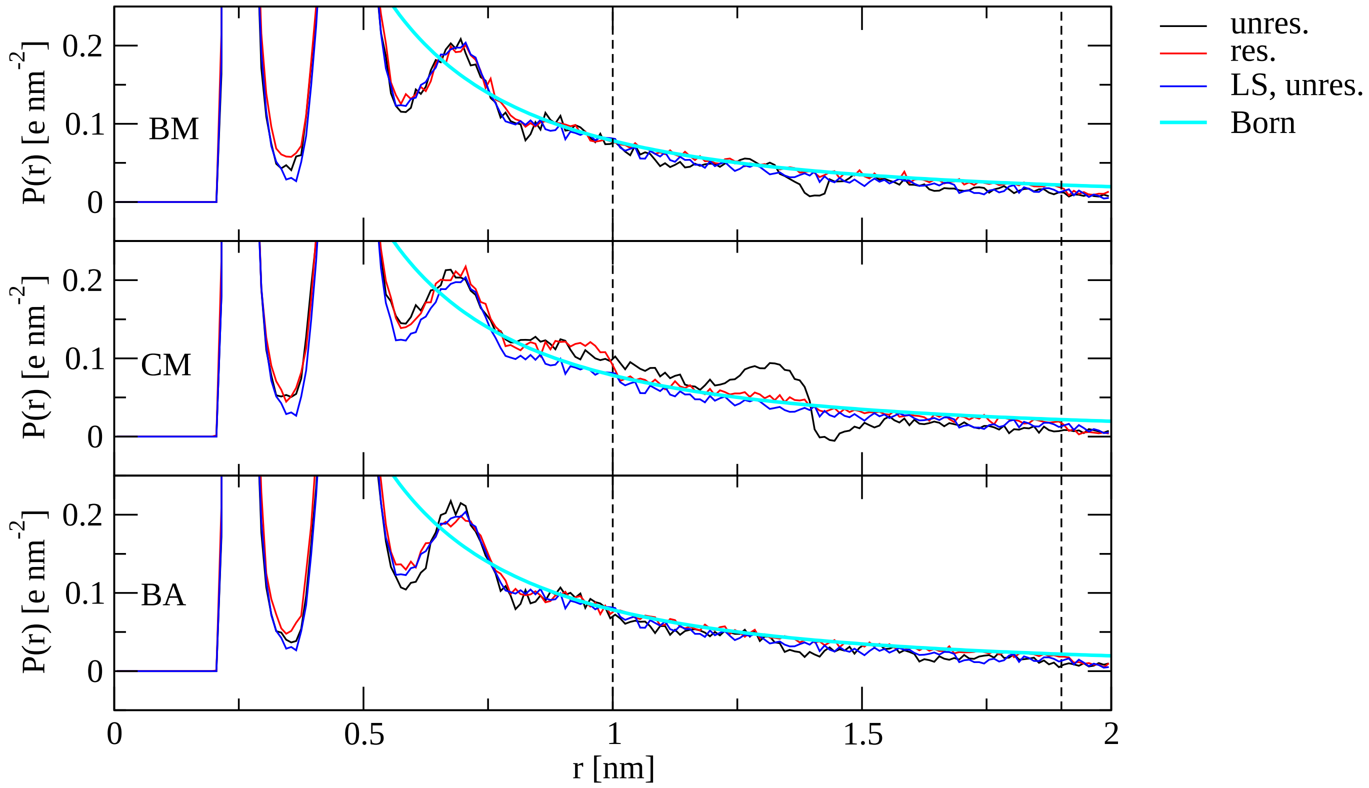

Figure S1: Radial polarization $P(r)$ (Eq. 44 of the main article) of water around the sodium ion for simulations without ("unres.") or with ("res.") a polarization restraint (Eq. 24 of the main article) during 1 ns simulations with the BM, CM and BA scheme, or with a LS electrostatic interaction function ("LS, unres."). The polarization restraint was applied in a spherical shell extending from 1.0-1.9 nm around the ion. All simulation settings were as described in Section III.1 of the main article. The LS scheme was applied using the particle-particle particle-mesh algorithm with tinfoil boundary conditions, a spherical hat charge-shaping function with a width of 1.0 nm, a triangular-shaped cloud assignment function, a finite-difference scheme of order 2 and a grid spacing of about 0.13 nm. The cyan line depicts the Born polarization (Eq. 18 of the main article). Dashed vertical lines indicate distances of 1.0 and 1.9 nm from the ion, *i.e.* the range where the polarization restraint was applied.

S.II Variation of force constant and decay time for the electrostatic potential restraint

Table S1 reports the average electrostatic potential $\langle \phi(\mathbf{r}_I) \rangle$ monitored at the ion site in simulations involving an electrostatic potential restraint (Eq. 9 of the main article) using different values for k and τ, namely $k = 125$, 250 or 500 kJ^{-1}·mol·e^2 and $\tau = 5$ or 50 ps. The data refers to simulations performed with the BM scheme for the treatment of electrostatic interactions. In an unrestrained simulation, $\langle \phi(\mathbf{r}_I) \rangle$ evaluates to -657.72 kJ·mol$^{-1} \cdot e^{-1}$, and the corresponding root-mean-square fluctuation (rmsf) evaluates to 43.65 kJ·mol$^{-1} \cdot e^{-1}$ (Table 1 of the main article).

The restraint to a target value $\phi_{tar} = -710.86$ kJ·mol$^{-1} \cdot e^{-1}$ is satisfied very well for decay times of 5 ps, regardless of whether k is set to 125, 250 or 500 kJ^{-1}·mol·e^2. For the investigated choices of force constants, combined with $\tau = 5$ ps, the rmsf of the electrostatic potential at the ion site is virtually the same. When the decay time is increased to 50 ps, the target value is satisfied less well, but is approached upon increase of the force constant. Note that throughout, the rmsf of the electrostatic potential is slightly larger in simulations with a larger decay time.

k [$\mathrm{kJ}^{-1}\cdot\mathrm{mol}\cdot e^2$]	τ [ps]	$\langle\phi(\mathbf{r}_I)\rangle$ (rmsf) [$\mathrm{kJ}\cdot\mathrm{mol}^{-1}\cdot e^{-1}$]
125	5	-709.71 (38.09)
125	50	-699.80 (42.57)
250	5	-709.62 (37.48)
250	50	-704.46 (41.73)
500	5	-710.33 (37.90)
500	50	-707.51 (41.47)

Table S1: Average electrostatic potential $\langle\phi(\mathbf{r}_I)\rangle$ and associated root-mean-square fluctuation (rmsf) at the sodium ion site, monitored during 1 ns simulation of a hydrated sodium ion with application of an electrostatic potential restraint (Eq. 9 of the main article) to the target value $\phi_{tar} = -710.86$ kJ·mol$^{-1} \cdot e^{-1}$ in a simulation with the BM scheme. All simulation settings were as described in Section III.1 of the main article, except for the force constant k and decay time τ, which were varied. The reported electrostatic potentials were calculated with the same electrostatic interaction function as used for configurational sampling.

S.III Variation of force constant and decay time for the polarization restraint

Figure S2 shows the radial polarization $P(r)$ monitored around the ion site in simulations involving a polarization restraint (Eq. 24 of the main article) using different values for k and τ, namely $k = 0.38$, 0.75 or $1.5 \cdot 10^7$ kJ·mol$^{-1} \cdot e^{-2}$·nm^4 and $\tau = 5$ or 50 ps.

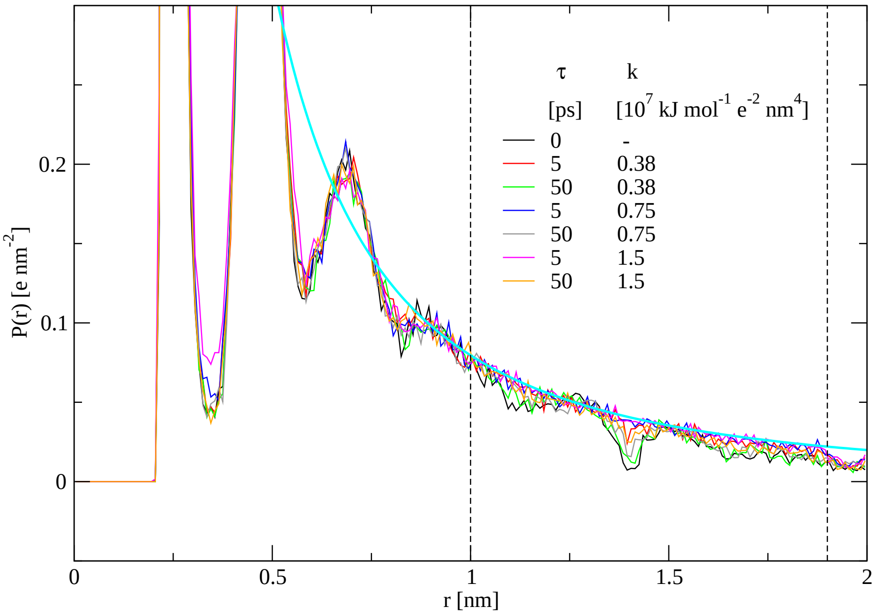

Figure S2: Radial polarization $P(r)$ (Eq. 44 of the main article) of water around the sodium ion for simulations without or with a polarization restraint (Eq. 24 of the main article) during 1 ns simulations with the BM scheme. The polarization restraint was applied in a spherical shell extending from 1.0-1.9 nm around the ion. All simulation settings were as described in Section III.1 of the main article, except for the force constant k and and decay time τ, which were varied. The cyan line depicts the Born polarization (Eq. 18 of the main article). Dashed vertical lines indicate distances of 1.0 and 1.9 nm from the ion, *i.e.* the range where the polarization restraint was applied.

The data refers to simulations performed with the BM scheme for the treatment of electrostatic interactions. In an unrestrained simulation, $P(r)$ has a dip at the cutoff distance $R_C = 1.4$ nm and is underpolarized beyond the cutoff distance in comparison to the target polarization (the Born polarization; Eq. 18 of the main article). The restraint to the Born polarization in a range extending from 1.0-1.9 nm from the ion is satisfied very well for decay times of 5 ps, regardless of whether k is set to 0.38, 0.75 or $1.5 \cdot 10^7$ kJ·mol$^{-1} \cdot e^{-2}$·nm^4 although a small dip can still be seen with the lowest force constant (red curve in Figure S2). When the decay time is increased to 50 ps, the target value is satisfied less well. However, it can be seen that the artifacts in the polarization are reduced upon increase of the force constant (green, gray and orange curves in Figure S2).

S.IV Charging free energy calculation

Table S2 provides the numerical values of the thermodynamic integration curves shown in Figure 4 of the main article along with the target electrostatic potentials $\phi_{tar}(q_i)$ employed in the simulations and the corresponding electrostatic potential corrections $\tilde{\phi}_{cor}(q_i)$ (Eq. 49 in Section III.3 of the main article). The electrostatic potential averages $\langle\phi(\mathbf{r}_I)\rangle$ at the ion site obtained from the unrestrained simulations were used as $\phi(q_i)$ in Eq. 49 of the main article to calculate $\phi_{tar}(q_i)$.

q_i [e]	$\langle\phi(\mathbf{r}_I)\rangle$ (unres.) [kJ·mol$^{-1} \cdot e^{-1}$]	$\tilde{\phi}_{cor}(q_i)$ [kJ·mol$^{-1} \cdot e^{-1}$]	$\phi_{tar}(q_i)$ [kJ·mol$^{-1} \cdot e^{-1}$]	$\langle\phi(\mathbf{r}_I)\rangle$ (res.) [kJ·mol$^{-1} \cdot e^{-1}$]
0.0	37.5	0.0	37.5	37.5
0.05	7.4	-2.7	4.7	4.4
0.1	-21.4	-5.3	-26.7	-26.4
0.2	-78.4	-10.6	-89.0	-88.9
0.3	-136.8	-15.9	-152.7	-152.6
0.4	-203.7	-21.3	-225.0	-224.5
0.5	-274.1	-26.6	-300.7	-300.5
0.6	-349.3	-31.9	-381.2	-381.1
0.7	-426.5	-37.2	-463.7	-463.6
0.8	-505.3	-42.5	-547.8	-547.4
0.9	-582.4	-47.8	-630.2	-629.4
1.0	-657.6	-53.1	-710.8	-709.6

Table S2: Solvent-generated electrostatic potentials $\langle\phi(\mathbf{r}_I)\rangle$ at a sodium ion site averaged over simulations of 1 ns length of charge states q_i of the ion in simulations without ("unres.") or with ("res.") an electrostatic potential restraint (Section III.3 of the main article). For the latter simulations, the target electrostatic potentials $\phi_{tar}(q_i)$ are also reported. They derive from the average electrostatic potential monitored in the unrestrained simulations in combination with electrostatic potential corrections $\tilde{\phi}_{cor}(q_i)$ (Eq. 49 in Section III.3 of the main article).
